# The power of phylogenetic approaches to detect horizontally transferred genes

**DOI:** 10.1186/1471-2148-7-45

**Published:** 2007-03-21

**Authors:** Maria S Poptsova, J Peter Gogarten

**Affiliations:** 1 Department of Molecular and Cell Biology, University of Connecticut, USA

## Abstract

**Background:**

Horizontal gene transfer plays an important role in evolution because it sometimes allows recipient lineages to adapt to new ecological niches. High genes transfer frequencies were inferred for prokaryotic and early eukaryotic evolution. Does horizontal gene transfer also impact phylogenetic reconstruction of the evolutionary history of genomes and organisms? The answer to this question depends at least in part on the actual gene transfer frequencies and on the ability to weed out transferred genes from further analyses. Are the detected transfers mainly false positives, or are they the tip of an iceberg of many transfer events most of which go undetected by current methods?

**Results:**

Phylogenetic detection methods appear to be the method of choice to infer gene transfers, especially for ancient transfers and those followed by orthologous replacement. Here we explore how well some of these methods perform using *in silico *transfers between the terminal branches of a gamma proteobacterial, genome based phylogeny. For the experiments performed here on average the AU test at a 5% significance level detects 90.3% of the transfers and 91% of the exchanges as significant. Using the Robinson-Foulds distance only 57.7% of the exchanges and 60% of the donations were identified as significant. Analyses using bipartition spectra appeared most successful in our test case. The power of detection was on average 97% using a 70% cut-off and 94.2% with 90% cut-off for identifying conflicting bipartitions, while the rate of false positives was below 4.2% and 2.1% for the two cut-offs, respectively. For all methods the detection rates improved when more intervening branches separated donor and recipient.

**Conclusion:**

Rates of detected transfers should not be mistaken for the actual transfer rates; most analyses of gene transfers remain anecdotal. The method and significance level to identify potential gene transfer events represent a trade-off between the frequency of erroneous identification (false positives) and the power to detect actual transfer events.

## Background

Horizontal gene transfer (HGT) is postulated to play an important role in evolution because sometimes the transferred genes allow the recipient to adapt to new ecological niches (e.g.: [1-4]). No type of gene appears to be immune to horizontal transfer [5]; however, most of the recently transferred genes appear to belong to a different pool of genes as compared to housekeeping genes [6-8]. Some transfers will undoubtedly provide a selective advantage to the recipient, for example those genes that allow the recipient to occupy a new ecological niche; however, many gene transfers appear to be selectively neutral and nearly neutral [7,9]. Even in the case of an orthologous displacement, where the transferred gene replaces an incumbent gene and is permanently fixed in the recipient lineage [10,11], the selective advantage for the recipient lineage is not always apparent, and the displacement at least in some instances might be a random process.

An assessment of the importance of HGT in microbial evolution in general, and in phylogenetic reconstruction in particular, depends in part on the frequency with which genes are transferred. A few phylogenetic misplacements due to HGT were recently suggested [7,12]; but many have expressed the opinion that these events will be the rare exception rather than the rule (e.g. [13,14]). The potential of HGT to create phylogenetic artifacts undoubtedly depends on the HGT frequency. Are the currently detectable transfers mainly false positives [15], or are they the tip of an iceberg of many transfer events most of which go undetected by current methods [1,16]?

The known methods of HGT detection can be divided into parametric and phylogenetic [17]. Parametric methods are based on the detection of atypical sequence composition for genome regions in comparison with a whole genome, whereas phylogenetic methods search for conflicts between the phylogeny inferred for a gene and the assumed organismal phylogeny. It appears that the different approaches detect largely non-overlapping sets of transfer events [18,19]. Because of sequence amelioration, parametric methods are limited to detect recent transfers [20], and their success hinges on donor and recipient having different sequence characteristics [21-23]. In contrast, phylogenetic methods are limited because they rely on homologous sequences being available from other organisms separating donor and recipient. Furthermore, other processes different from HGT can give rise to incongruence between gene and presumed organismal phylogeny.

**Figure 1:**
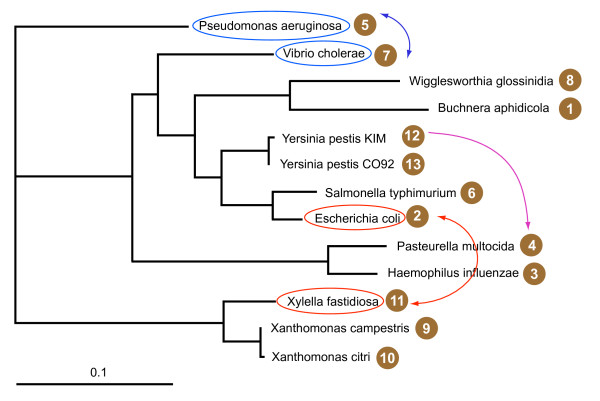
**Example of Artificial Transfers between Species**. The topology of the depicted tree was calculated from 236 gene families using the majority-rule consensus method; branch lengths were calculated from 16S rRNA. The tree should be considered as unrooted. To calculate SPR distances (Figure 2C) the tree was considered as rooted in the branch leading to (*Xylella fastidiosa*, *Xanthomonas campestris *and *X. citri*). The double-headed arrows indicate the *in silico *exchanges analyzed in Figure 3.

**Figure 2:**
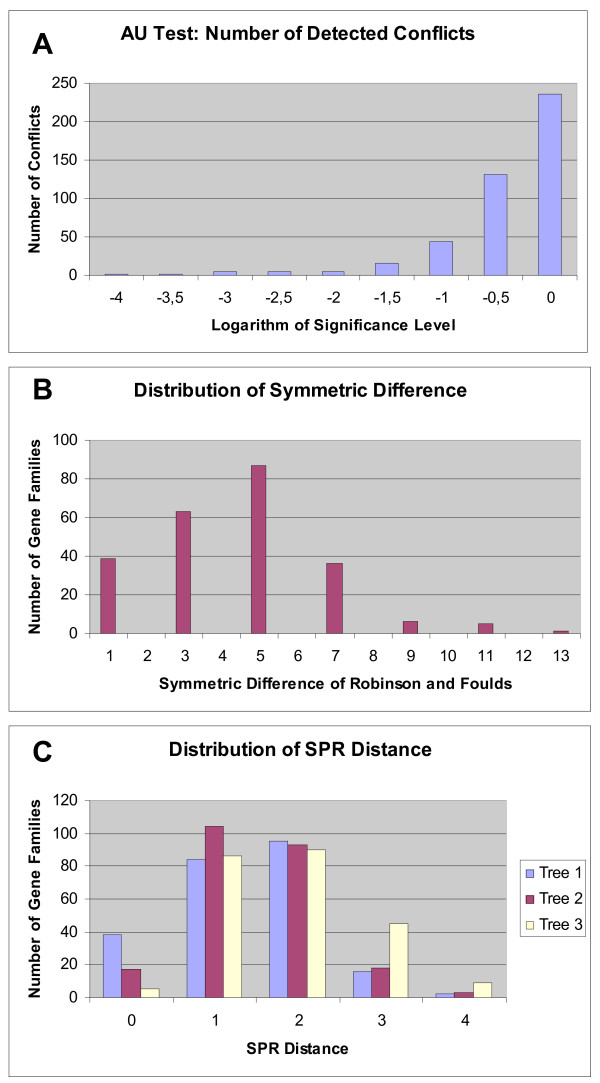
**Comparison of the gene phylogenies present in the original dataset**. Panel A gives the distribution of AU-test results. The AU-test was applied to the original dataset, and the distribution of AU-test results is depicted as a function of the significance level. Histogram bars give the number of all gene families that were different from the consensus tree (Figure 1) at the indicated significance level. Panel B gives data for the Symmetric Difference of Robinson and Foulds [27]. Tree distances were calculated between the consensus tree and each individual gene tree. The mean distance between gene tree and consensus is 3.37 with the standard deviation of 2.32. Panel C gives the number of edits (SPR events corresponding to HGTs) calculated according to [56]. To illustrate that the results do not depend on a particular reference tree, we used three different trees: Tree 1 is the consensus tree depicted in Figure 1, tree 2 and 3 are the trees that together with tree 1 were reported as showing the least amount of conflict with the individual gene phylogenies (trees 2 and 3 in Figure 2 of [25]).

Becerra and collaborators introduced *in silico *transfer to test the efficiency of parametric methods [23]. Here we extend their approach by applying phylogenetic methods to detect *in silico *HGTs. As a test case we used orthologous gene families from 13 proteobacterial genomes (Figure 1) that were shown by different approaches to contain only few detectable HGT events [24,25]. It is possible that this dataset contains few, or many, undetected gene transfer events; certainly the individual gene phylogenies are not all congruent with one another (Figure 2). The conflicts with the reference phylogeny could either be due to the evolutionary histories of genes actually being different, or, more likely, due to the limited amount of phylogenetic information present in the individual gene phylogenies. We chose this dataset because it provides a realistic backdrop against which to detect additional *in silico *transfer events. We test three approaches for their efficiency to detect HGT events: the AU test [26], the Symmetric Difference, or Robinson and Foulds distance [27] and Bipartition spectra or Lento plots [24,28].

The AU test, or *approximately unbiased *test [26], is based on the confidence of phylogenetic tree selection. For each tree tested on a dataset, the AU test estimates the probability that the tree might be the true tree describing the history of the dataset under consideration. The greater the *P*-value produced by AU test, the greater the probability that the tested tree is the true tree [26]. The AU test has been designed for obtaining the confidence set of trees using maximum-likelihood. Not only one best tree should be considered, but all the trees from a confidence set, i.e. trees with P-value higher than a significance level alpha. The trees that are not included in the confidence set are rejected. The smaller the P-value for a given tree, the more certain can this tree be rejected as reflecting the history of the dataset. If a dataset rejects the organismal phylogeny with significance level alpha, this dataset is considered incompatible with the organismal phylogeny, and one of the reasons for this incompatibility is HGT, although in the analysis of real gene families other reasons for incompatibility, for example unrecognized paralogy, need to be considered as well. The significance level gives the probability that the dataset erroneously is considered incompatible with the organismal tree.

Using the similar SH test [29] in an analysis of 13 gamma proteobacteria only few gene phylogenies were determined as incompatible with the consensus phylogeny [25]. The authors' concluded that therefore gene transfer should be considered rare [25]. See [30] for a controversial discussion of this assertion.

Bipartition spectra or Lento plots break the phylogenetic information contained in a dataset into small quanta of information. The Lento plot [28] was adopted to comparative genome analyses [24] by giving the number of gene families that support a bipartition, and the number of gene families that support a conflicting bipartition. Two conflicting bipartitions cannot coexist on the same bifurcating tree. Advantages of bipartition analyses are that a genome wide consensus (the plurality bipartitions) can be extracted without combining genes into a single dataset, and that individual splits or bipartitions are considered and not the whole gene phylogeny. Gene families that at a chosen level of support conflict with one or more of the plurality bipartitions are considered incompatible and as candidates for HGTs. The case of orthologs from the 13 gamma proteobacterial genomes is particularly useful for this approach, because the dataset contains eight bipartitions supported by the majority of gene families, whereas for other groups of bacteria the number of bipartitions with majority support is much smaller [24].

Finally, we used the difference between organismal history and the individual gene phylogeny as a measure of incompatibility between two trees. The Symmetric Difference, or Robinson and Foulds distance[27] gives the number of bipartitions that are present in one tree and absent in the other. In contrast to the Lento plot approach, this metric does not take into account the statistical significance of individual branches. To obtain a significance value we used the distribution of distances between the gene and the organismal phylogeny before the *in silico *transfer (Figure 2B). To test the different approaches we performed a series of *in silico *experiments simulating gene transfers between species for a well-studied (e.g., [24,25,30]) set of 13 gamma-proteobacteria.

## Results and discussion

For a set of 13 gamma proteobacetria 236 gene families were assembled using the strict reciprocal top scoring BLAST hit method (see Methods for details). For each gene family a maximum likelihood tree was reconstructed. The tree depicted in Figure 1 was calculated from 16S rRNA sequences using maximum likelihood [31]. Its topology is identical to the majority-rule consensus tree [32] calculated from all individual gene family trees. In the *in silico *experiments, two types of transfer were simulated: exchange of genes between two species and donation of a gene by one species to another. Exchange of genes between two species simulates a transfer that occurred somewhere along the two terminal branches leading to the two extant species, donation of a gene by one species to another represents a recent transfer that leads to two organisms having identical sequences, and both of these organisms are included in the analysis. In case of the exchanges, some of the genome pairs will simulate more ancient transfers than others (e.g., *Yersinia pestis *with *E. coli*, *versus Pseudomonas aeroginosa *with *Vibrio cholerae*) depending on the lengths of the terminal branches. In case of the donation, all of the transfers simulate recent events. All possible transfers were simulated for each gene tree and for each pair of species. Each gene tree with *in silico *exchange was compared against the consensus tree (Figure 1) using three different approaches: AU test [26], Symmetric Difference of Robinson and Foulds [27] and Bipartition Spectra [24].

### AU test

The AU test, or approximately unbiased test, assesses the confidence of phylogenetic tree selection [26]. The AU test estimates the probability that a given tree is the true tree according to which a dataset in question was generated. For each family of orthologous genes we test, if the gene family could have been generated according to the organismal phylogeny without any gene transfer. As organismal tree we use the consensus tree calculated from the individual gene trees using the majority-rule consensus method (see Methods). For each gene family, the P-value determined by the AU test for the consensus tree corresponds to the probability of identifying the gene family as having evolved according to the consensus (i.e., the null hypothesis is that the gene family evolved according to the consensus). When the SH test was applied to the proteobacterial genomes without *in silico *transfers only two significant conflicts (with a significance level of 5%) with the assumed species phylogeny were reported [25]. We obtained similar results by applying the AU test to the orthologous gene families from the gamma proteobacterial genomes (see Methods for the selection of gene families). The distribution of AU-values is presented in Figure 2A. Only two families out of 236 showed a conflict at the significance level of 5 *10^-4^, 5 conflicts were found at the significance level of 0,01 and 26 conflicts at the significance level of 0,05.

**Figure 3:**
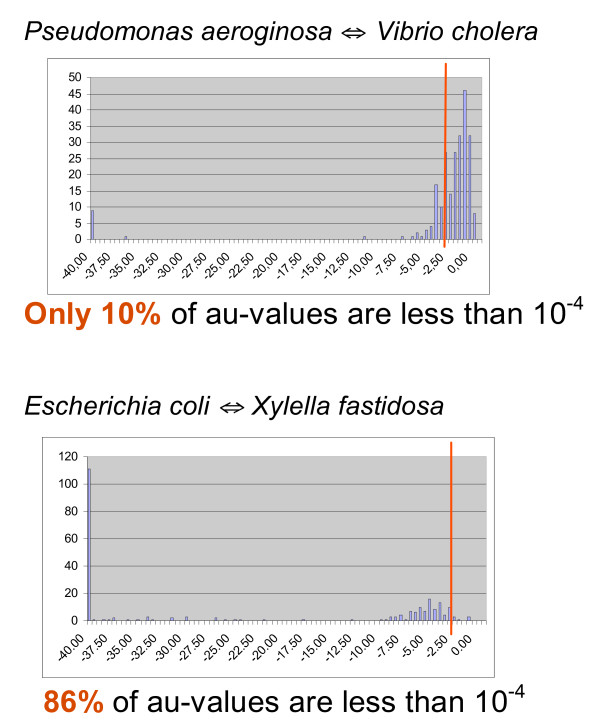
**Distribution of AU test results for two cases of *in silico *HGT**. The figures show the distribution of the logarithms of AU test values for an *in silico *gene exchange between two genome pairs. The red line corresponds to the p-value of 10^-4^.

**Figure 4:**
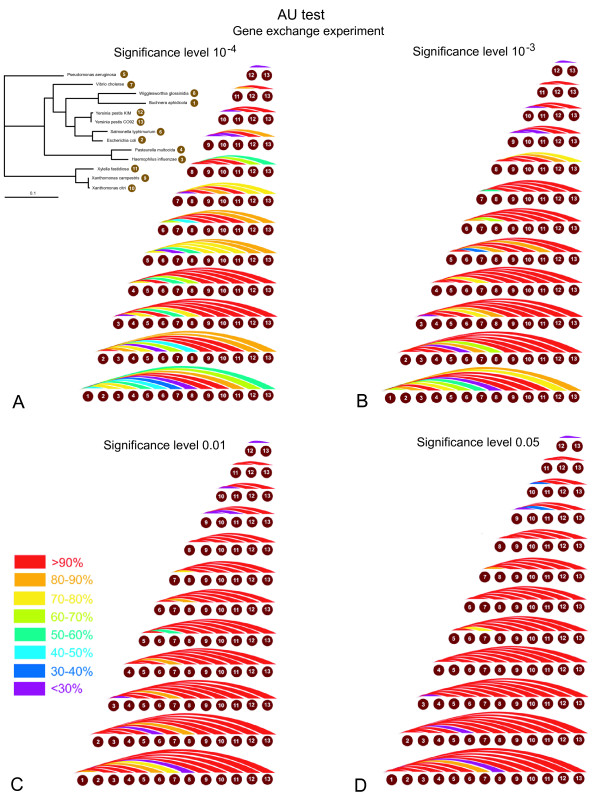
**Power of HGT detection for *in-silico *gene exchanges using the AU-test at different significance levels**. The power of detection is calculated as the percentage of gene families that were detected as significantly different from the consensus using the indicated significance levels. Each colored arc corresponds to one genome pair. The arc is colored according to the percentage of genes that after the swap resulted in a significant conflict with the consensus. Panels A, B, C and D show results for significance levels of 10^-4^, 10^-3^, 10^-2^, and 0,05, respectively.

Two examples of *in silico *exchange of genes between two species are shown on Figure 1. Histograms of the distribution of AU test values for all gene families for the two cases of *in silico *gene exchange are presented on Figure 3. The two transfers yield significant conflicts with very different frequencies. In case of a gene exchange between *Pseudomonas aeroginosa *and *Vibrio cholera *only 12% of gene families produced P-values less than 10^-4 ^when compared with the species tree. The power of detection increases when the species undergoing the gene swap are separated in the tree by a larger number of nodes. In case of the exchange between *Escherichia coli *and *Xylella fastidiosa *the number of families with significant conflict (P < 10^-4^) is 94 %. The power of detection for all possible pairwise swaps with four different significant levels is summarized in Figure 4. Tables showing exact detection levels and reflecting the tree topology are included in the additional files [Additional file 1].

**Figure 5:**
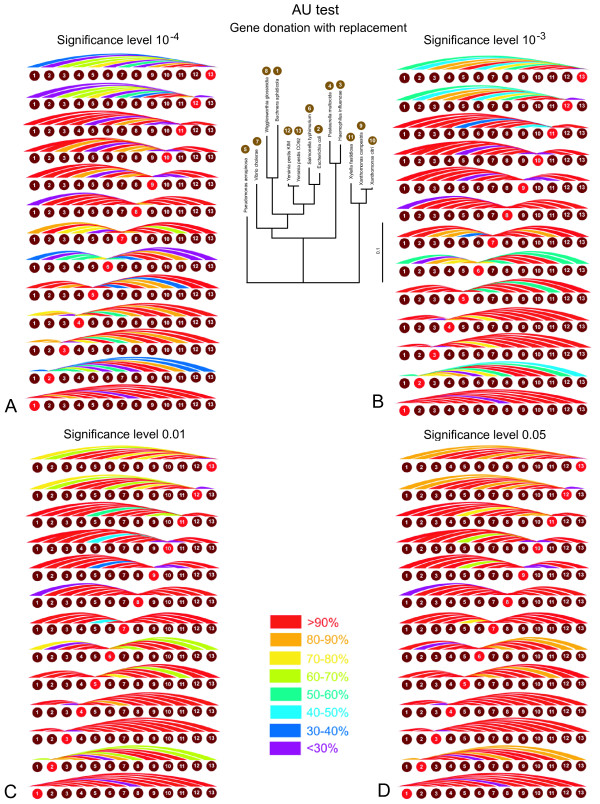
**Power of HGT detection for *in-silico *gene donations using the AU-test at different significance levels**. The power of detection is calculated as the percentage of gene phylogenies that were detected as significantly different from the consensus using the indicated significance levels. Each colored arc corresponds to an event where a gene is transferred from one species to another, and the existing gene was removed from the recipient. The arc is colored according to the percentage of genes that after the swap resulted in a significant conflict with the consensus. Panels A, B, C and D show results for significance levels of 10^-4^, 10^-3^, 10^-2^, and 0,05, respectively.

In another series of experiments we simulated recent HGT by donating a gene from one species to the other, as depicted by the unidirectional arrow in Figure 1 designating transfer from *Yersinia pestis KIM *to *Pasteurella multocida*. The donated gene replaces the existing ortholog so that the new dataset contains two identical sequences. We simulated all possible gene donations to all species, and for each compare the new tree with the reference tree. The power of detection with four different significant levels is presented in Figure 5. Tables showing exact detection levels and reflecting the tree topology are included in the additional files [Additional file 1].

An advantage of the AU test is that its significance level directly relates to the number of expected false positives (i.e., the probability that a conflict is inferred in error due to chance). However, the significance level does not consider systematic artifacts and biases generated in phylogenetic reconstruction. Furthermore, the significance level does not inform the user on the number of false negatives. The latter can be assessed through either simulations or *in silico *transfers. Internal branches of a phylogenetic tree frequently are difficult to reconstruct with confidence. It is therefore reasonable to assume that transfers and exchanges between terminal branches of a phylogenetic tree (that are separated by more than one node) are easier to detect than transfers that occurred earlier in evolution. Our use of *in silico *transfers thus likely under estimates the number of false negatives encountered in the analyses of real data; however, a verification of this expectation will require simulation or *in silico *transfers that swap whole clades, and not individual sequences only. For the experiments performed here on average, and excluding exchanges between sister taxa, the AU test at a 5% level detects 90,3% of the transfers and 91% of the exchanges as significant. At a significance level of 10^-4^, which would be more appropriate considering that multiple test are performed (see below), the detection rate drops to 71 % and 70% respectively. We conclude that an individual gene family frequently does not contain sufficient phylogenetic information to detect HGT events reliably.

A disadvantage of the AU test with respect to HGT-detection is that it requires the knowledge of the organismal tree that one usually doesn't have. The studies of Lerat et al. [26], where the SH test were applied to only 5 possible organismal trees, were extended to 105 not significantly different "true" trees [30], and both, SH and AU tests were applied. The number of rejected trees varies considerably over the range of 105 trees, thus questioning the statement that the gamma proteobacterial core is free from HGT. The choice of the "best" tree is still difficult because all these trees are not significantly different from each other (see discussion on the choice of the best tree in [33]).

### Symmetric difference of Robinson and Foulds

The Symmetric Difference of Robinson and Foulds [27] between two trees gives the number of bipartitions that are different in two trees, or the number of bipartitions or splits that are in one tree and not in the other. The distribution of the symmetric difference values for the original non-swapped data is depicted on Figure 2B.

**Figure 6:**
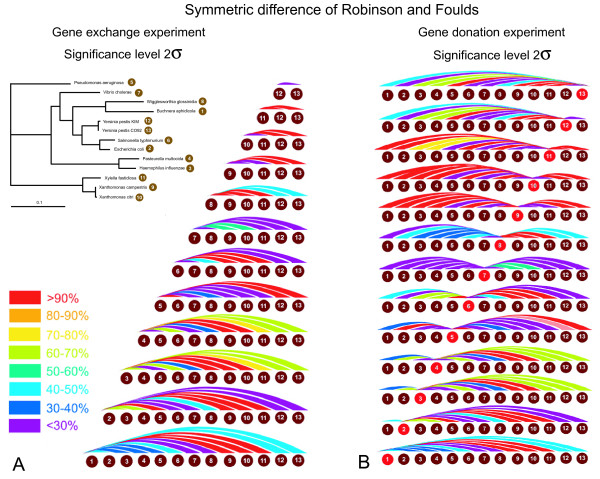
**Power of HGT detection using the symmetric difference of Robinson and Foulds distance (2 sigma significance level)**. The power of detection is calculated as the percent of gene families whose maximum likelihood tree after *in silico *transfer has a distance to the consensus tree that is more than two standard deviations larger than the mean of distances for all gene families before the *in silico *transfers. Each colored arc gives the results for one genome pair. The colors denote the percentage of genes that after the swap resulted in a significant conflict with the consensus. Panel A gives results from gene exchange experiments, and Panel B contains results from the experiment of gene donation with replacement.

We use the mean and standard deviation of the distance distribution in the original data to assess the significance of a distance after *in silico *transfer. We considered a distance to the consensus as significant, if this distance was at least two standard deviations larger than the mean of the distance distribution of the non-swapped original data. In case of a normal distribution, this cutoff level corresponds to a significance level of 2.5%. The results from all possible pairwise swaps are shown in Figure 6A, and the results of all possible gene donations with replacement from one species to another are shown on Figure 6B. Tables showing exact detection levels and reflecting the tree topology are included in the additional files [Additional file 2].

This rather simple minded approach to evaluate the significance of the distance between two trees was surprisingly effective, but clearly inferior to the AU-test at comparable significance levels. Ignoring transfers between sister taxa on average 57.7% of the exchanges and 60% of the donations were identified as significant conflicts. This method could greatly be improved, if it were to consider the support values of the bipartitions not shared between the trees. This would make this approach more similar to the Lento plot analysis (see below). One also could use more complex distance measures. The latter approach was implemented in [34] using a distance measure calculated from the symmetric distance of Robinson and Foulds [27] combined with the maximum agreement subtree [35].

### Analysis of bipartition spectra

**Figure 7:**
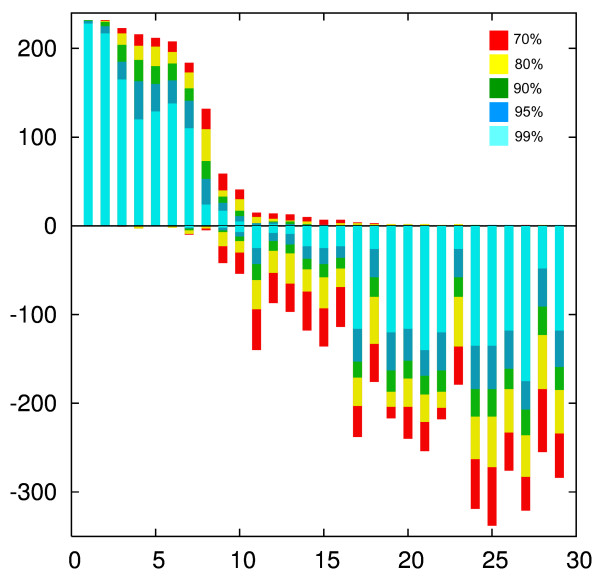
**"Lento"-plot [28] depicting the phylogenetic information retained in 13 Gamma proteobacterial genomes**. This plot is a modified summary of the analyses and results reported in [24]. The plot summarizes the phylogenies from protein families that were determined to have putative orthologs in each of the 13 genomes using the reciprocal best hit criterion [47, 52]. 29 bipartitions (from a total of 4082) were found to be supported by at least one gene family with more than 70% bootstrap support. The bipartitions are ranked in order of the number of supporting families at the 70% bootstrap support level. For each bipartition the bars in the positive direction give the number of gene families that support the bipartition with the indicated (color coded) support value, the bars in the negative direction give the number of supported conflicting bipartitions found in all of the gene families. This number can be greater than the number of gene families, because a single gene family can support several conflicting bipartitions. Note that the first eight bipartitions are supported by the majority of gene families, and that only three dataset conflict with these plurality bipartitions at the 99% bootstrap support level. See [24] for further discussion.

Bipartition spectra (also known as Lento plots) focus only on bipartitions that have statistical support. Here we use bootstrap support values calculated using maximum likelihood trees. The majority of the gene families strongly support eight bipartitions. We call these bipartitions majority bipartitions. Bipartition analysis of the original data showed that only very few gene families provide high bootstrap support for bipartitions conflicting with the majority bipartitions ([24], Figure 7 and Table 1). This finding is in accord with previous analyses that suggested that the 13 gamma proteobactrial genomes used in this study contain only few gene families in significant conflict with the consensus [25]).

**Table 1:** List of 13 gamma proteobacteria species

1.	Buchnera aphidicola str. Bp (Baizongia pistaciae)
2.	Escherichia coli CFT073
3.	Haemophilus influenzae Rd KW20
4.	Pasteurella multocida subsp. multocida str. Pm70
5.	Pseudomonas aeruginosa PAO1
6.	Salmonella typhimurium LT2
7.	Vibrio cholerae O1 biovar eltor str. N16961
8.	Wigglesworthia glossinidia endosymbiont of Glossina brevipalpis
9.	Xanthomonas campestris pv. campestris str. ATCC 33913
10.	Xanthomonas axonopodis pv. citri str. 306
11.	Xylella fastidiosa 9a5c
12.	Yersinia pestis KIM
13.	Yersinia pestis CO92

Thirteen complete genomes from gamma-proteobacteria were downloaded from the ncbi ftp-site [50] on July 2005. All of the analyses reported here were performed on the encoded protein sequences.

**Figure 8:**
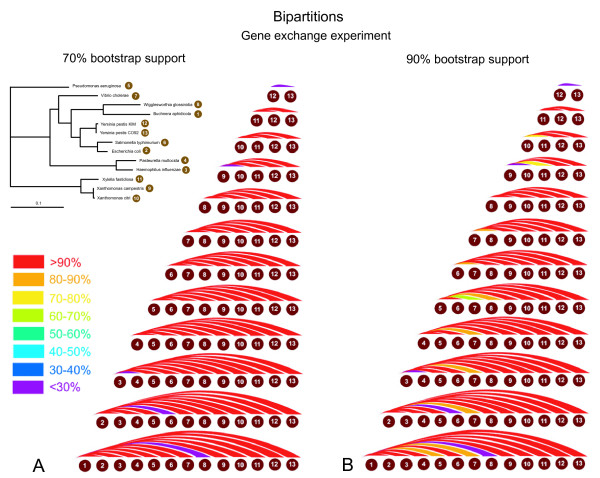
**Power of HGT detection using bipartition spectra on genome data with *in silico *transfer**. Power of detection is calculated as the percentage of gene families that after an *in silico *transfer supported one or more bipartitions with more than 70% (panel A) or 90% (panel B) bootstrap support that were in conflict with one of the eight plurality bipartitions (see Table 2). Each colored arc corresponds to one genome pair, and the percent of gene families with detected conflict is color coded.

The results for detection of *in silico *gene exchange between species are depicted in Figure 8. Tables showing exact detection levels and reflecting the tree topology are included in the additional files [Additional file 3]. At the 70% bootstrap support level (Figure 8A), most *in silico *transfers resulted in at least one conflict with one of the majority bipartitions. This finding was unexpected, because the eight plurality bipartitions correspond to an unresolved tree including one node with eight emerging branches [24]. However, only in case of the five sister species are the leaves not separated by at least one of the bipartitions with majority support. The five instances of sister species result in 5 genome pairs for which the power of HGT detection using this approach is zero (purple arcs in Figure 8): 1–8 (*Buchnera aphidicola *and *Wigglesworthia glossinidia*), 2–6 (*Escherichia coli *and *Salmonella typhimurium*), 3–4 (*Haemophilus influenzae *– *Pasteurella multocida*), 9–10 (*Xanthomonas campestris *and *Xanthomonas axonopodis*) and 12–13 (*Yersinia pestis KIM *and *Yersinia pestis CO92*) (see Figures 1 and 8). In all other instances the power of detection was on average 97% using a 70% cut-off for the conflicting bipartitions (Figure 8A). If the cut-off level for conflicting bipartitions is increased to 90% (Figure 8B), the rate of detection drops to an average of 94.2%, but overall, the level of detection compares favorably to the one obtained with the AU test.

The experiment of gene donation with replacement for bipartitions is quite time consuming: for each new tree topology, resulting from a gene donation, one must generate and analyze 100 bootstrapped replicates, which on the University of Connecticut's biocluster (PowerPC G5 2.3 GHz, 2GB RAM) using phyml takes about 20 minutes for 100 bootstrap replicates; multiplying by 236 families gives a computation time of about 3 days for one gene donation experiment for one genome. For the set of 13 species, there are 13*12 = 156 possible donations, thus it would take about 156*3 = 468 days of computational time. However, a gene donation with replacement results in two identical sequences being present in the dataset. It is reasonable to assume that these two identical sequences will form a highly supported group. We therefore estimate the power of detection of the bipartition approach by determining the number of majority bipartitions (Table 2 and Figure 7) that conflict with the bipartition created through the gene transfer. The results of these comparisons are presented in Table 3. In our case the number of conflict can range from 0 to 8, but even one conflict is sufficient to detect the conflict created through HGT. Only the transfers between sister species evade detection through the bipartition approach.

**Table 2:** Bipartitions that are shared by the majority of the families in the original dataset.

**bipartition**	**% of families that support bipartition**	**Number of families with conflicting bipartition at**				
		**70%**	**80%**	**90%**	**95%**	**99%**
...........**	98	0	0	0	0	0
........***..	98	0	0	0	0	0
..**.........	94	1	0	0	0	0
........**...	92	3	3	0	0	0
.*...*.......	90	0	0	0	0	0
*......*.....	88	2	2	0	0	0
....*...***..	78	10	9	5	3	2
*....*.....**	56	5	3	0	0	0

Numbers give the percent of gene families that support the indicated consensus bipartitions with more than 70% bootstrap support, and the number of gene families that support at least one conflicting bipartition with more than 70% bootstrap support. Bipartitions are indicated in the style used in PHYLIP [32], the order of genomes is as listed in Table 1.

**Table 3:** Bipartitions: Gene Donation with Replacement Experiment.

	**1**	**2**	**3**	**4**	**5**	**6**	**7**	**8**	**9**	**10**	**11**	**12**	**13**
**1→**		3	2	2	2	3	1	0	4	4	3	3	3
**2→**	3		3	3	3	0	2	3	5	5	4	2	2
**3→**	2	3		0	2	3	1	2	4	4	3	3	3
**4→**	2	3	0		2	3	1	2	4	4	3	3	3
**5→**	2	3	2	2		3	1	2	2	2	1	3	3
**6→**	3	0	3	3	3		2	3	5	5	4	2	2
**7→**	1	2	1	1	1	2		1	3	3	2	2	2
**8→**	0	3	2	2	2	3	1		4	4	3	3	3
**9→**	4	5	4	4	2	5	3	4		0	1	5	5
**10→**	4	5	4	4	2	5	3	4	0		1	5	5
**11→**	3	4	3	3	1	4	2	3	1	1		4	4
**12→**	3	2	3	3	3	2	2	3	5	5	4		0
**13→**	3	2	3	3	3	2	2	3	5	5	4	0	

The entries indicate the number of conflicts between the bipartitions created through the transfer (i.e. the two identical sequences) and the eight majority bipartitions from Table 2. The donating genome is indicated on the left side, the receiving genome on top. See Figure 1 for the numbering of the genomes.

### Organismal phylogeny

The phylogeny used as a reference tree (see Figure 1) groups the two endosymbionts *Buchnera aphidicola *and *Wigglesworthia glossinidia *together. The monophyly of these and other insect endosymbionts was supported by several studies that were based on analyses of the available genome sequences [25,36,37]. However, the endosymbionts are characterized by reduced, AT rich genomes, and the placement of these sequences thus might reflect shared bias and not shared ancestry. It should be noted that the analysis reported here, as well as the studies reported in [25,36,37], used amino acid sequences; however, it is possible that the nucleotide bias might also impact the amino acid based studies. A recent study based on a non equilibrium model suggested that *Buchnera *and *Wigglesworthia *might not be monophyletic [38]. This study was based on a nucleotide sequence alignment of only two gene families, but it included 8 endosymbiotic gamma proteobacterial taxa. If the monophyly of *Buchnera *and *Wigglesworthia *were indeed an artifact due to shared bias it would indicate that even high support values for protein based phylogenies between not very divergent organisms have to be regarded with more skepsis than they usually receive. However, this finding would not detract from the findings and the value of this study, i.e., demonstrating that individual genes frequently contain too little information to decide between alternative phylogenies.

### Orthologous replacement *versus *"real" gene transfer

The use of phylogentic information to detect horizontally transferred genes restricts analyses to families of orthologs. In case of the 13 gamma proteobacterial genomes used in the study the assembled families of orthologs represents only about 7% of the studied genomes. These families of orthologous proteins represent genes under strong purifying selection, which tend to be transferred infrequently [1,39,40]. Most of the transferred genes identified through the comparison between strains do not have recognizable orthologs in divergent organisms; many of them, the so-called ORFans, do not have homologs in the current databanks at all. These genes are acquired from phage and plasmids, and not directly from other genomes [6,7,41,42]. While gene families with recognizable orthologs do not represent the typical transferred gene, the rate of transfer for the conserved, infrequently transferred genes is important, because these genes are used to reconstruct organismal phylogenies, and if transferred genes are not excluded from these analyses, the resulting phylogenies might represent neither the phylogenies of an individual gene nor the history of the organism [7].

### Correction for multiple tests

The analyses presented in this study, and the reported significance values are based on the individual gene phylogeny only and do not include a correction for multiple tests, as is the case in most phylogenetic analyses screening genome scale data for phylogenetic conflict (e.g. [43,44]). In case of the AU tests, it is straight forward to apply the Bonferoni correction [45], i.e., a significance level of (alpha/(number of parallel tests)) for the individual test gives the probability (alpha) that a result of any of the performed multiple tests might be considered significantly different due to error. Applying this correction would lead to even fewer of the transferred genes being detected. For an overall error probability of 5%, the individual test would need to apply a significance level of .02% (5% divided by the number of gene families tested) or 2*10^-4 ^(compare Figures 4A, 4B, 5A, and 5B). In case of the bipartition analyses a correction for multiple tests has not been established. Bootstrap support values for a clade provide a measure for the amount of information present in the analyzed data supporting this clade. Bootstrap support values were shown to be more conservative than probabilities (e.g., [46,47]). In a hypothesis testing framework, 100 minus % bootstrap support can be considered as a measure for identifying the clade as monophyletic in error (see [48] for a recent discussion). However, without correction the resulting error probabilities are vastly overestimated. For example, the Lento plot depicted in Figure 7 shows that at most 10 datasets conflict with the majority bipartitions with more than 70% support, and only five families conflict with one of the plurality bipartitions with more than 90% support. These numbers are far below the 70 (30% of the families) or 23 (10%) families that are expected per bipartiton, if 100% bootstrap support were equal to the error probability. Considering that at least some of the conflicts are due to HGT, and that other systematic artifacts are likely to have increased incongruence between individual gene phylogenies, the lack of conflict is even more testament to the conservative nature of the bootstrap support values.

### Comparison of different approaches

The AU test provides a good statistical framework to assess significance levels, i.e., the probability that a conclusion of incongruence is made in error. However, in assessing the phylogenies as a whole, many of the swapped or transferred genes evade detection. Using the fraction of detected *in silico *transfers as measure, the bipartition based analysis appears most successful in this test case; however, this finding cannot be generalized (see discussion below). We do not know the "real" rate of false positives, however, the significance levels and the total number of inferred transfers provide estimations and upper bounds for the number of false positives, respectively. Bootstrap support values for an individual split often are more conservative than probability estimates [46-48]. Figure 7 and Table 2 illustrate that using bipartitions that are supported by 90 % the rate of false positives over all bipartitions is less than 5/236 or 2.1%. (Only five gene families, i.e. 2.1%, showed conflicts with the plurality bipartitions, and most of these families probably represent real instances of gene transfer. The 2.1% of gene families with significant conflict thus provide an upper bound, the case that all these conflicts were identified erroneously, for the rate of false positives.) The success of the bipartition based approach probably is due to its focus on individual well supported bipartitions. In contrast, the AU test assesses all splits in a tree simultaneously. This might lead to changes in one part of the tree being masked by uncertainty in other parts of the tree. It also could be argued that the significance level in the AU test overestimates the false positive rate; for example the actual rate of false positives in case of the AU test performed with a 5% significance level might be much smaller than 5%. However, the data in Figure 2A suggest that the significance level is a reasonable estimator of the false positive rate.

The advantage of the Lento plot based approach is that gene transfer events can be detected even in the absence of a completely resolved organismal phylogeny. However, the power of detection of this approach depends on at least a few well-supported bipartitions being present in the majority of gene phylogenies. If such a majority signal can be extracted from the individual gene phylogenies, then the bipartition based approach can identify families that are likely candidates for gene transfer. If only few majority bipartitions are present in a bipartition spectrum, as is frequently the case in analyses of many genomes and with phylogenies that contain short internal branches, then the AU-test provides an alternative method to identify individual conflicting genes, provided a reasonable hypothesis for the organismal phylogeny can be developed. In case a reliable reference or consensus phylogeny is unavailable, other approaches that break down gene phylogenies into smaller units of phylogenetic information, e.g., quartet decomposition [49], might provide an alternative to the AU test.

The choice of method also is contingent on the reason why one wants to identify putatively transferred genes. If one is interested in case studies of transferred genes, then the AU test with a high significance level, or a bipartition spectrum both are effective in identifying candidate genes. However, if one wants to remove gene families from a genome based phylogenetic analyses that might taint a combined dataset, then the AU test with a 5% significance levels provides a workable solution, even though at this significance level one expects to remove about 5% of the gene families erroneously. Finally, at present none of the available approaches appears entirely satisfying to quantify rates of orthologous replacement; one needs to balance unacceptable high rates of false negatives against the reliability of assessing rates of false positive, and both types of errors need to be explicitly considered in the quantification.

## Conclusion

The existing phylogenetic methods of HGT detection should be treated with caution when attempting to estimate rates of transfer. The AU test provides a good measure of reliability with respect to false positives, but the rate of false negatives was high when stringent significance levels were applied. Decreasing the significance level, as expected, leads to a decrease in the false negative rate; with a 5% significance level the power of detection was 90% on average.

Bipartition spectra were surprisingly powerful in our test case. Even at a cut-off level of 70% only ten conflicts were detected in the original data, and many of these conflicts apparently correspond to real gene transfers [24,25]. At the lower cut-off level detection rates were better than 97% on average, and the rate of detection remained high, even when the cut-off level was increased. However, this success of the bipartition spectra cannot be generalized. For the 13 gamma-proteobacteria used in this study we have eight highly supported bipartitions, and most of the tested *in silico *transfers cross at least one of these consensus bipartitions. The power of the bipartiton based approach will decrease with the number of consensus bipartitions.

## Methods

Thirteen complete genomes from gamma-proteobacteria were downloaded from the ncbi's ftp-site [50] on July 2005. All of the analyses reported here were performed on the encoded protein sequences. The genomes used in this study are listed in Table 1.

236 families of putatively orthologous genes were detected using the strict reciprocal best Blast hit method [46,47,51,52] with an E-value cutoff of 10^-4^. Gene families were aligned with ClustalW version 1.83 [53] using default parameters. Special treatment of regions with ambiguous alignment was not necessary, because the orthologous sequences, selected by the strict reciprocal Blast hit method, were on average 61% identical with the standard deviation of ± 18%. For each family a maximum likelihood tree was calculated by Phyml [31] using the JTT model, four relative substitution rate categories, and an estimated gamma distribution parameter. The tree depicted in Figure 1 was calculated as consensus tree from the individual gene trees using the majority-rule consensus method with the program CONSENSE from the Phylip package [32]. The same phylogeny was obtained from a 16s RNA alignment using maximum likelihood as implemented in Phyml [31] using the HKY model, estimation of invariant sites, and among site rate variation described by a gamma distribution with estimated shape parameter.

### AU test

The p-values of the approximately unbiased test were calculated with the program Consel [54]. Log-likelihoods were estimated with Codeml from the PAML package [55]. The artificial swaps between genomes were simulated by changing places of the pair of genomes in multiple alignment files and in the reconstructed trees. Gene donation events were simulated by replacing a recipient genome sequence with a donor sequence in the alignment file, so that alignment file contains two identical sequences from the donor genome. The tree was modified by moving the branch of the recipient genome next to the donor genome. Each gene tree with simulated transfers was compared against the organismal tree, and those whose AU test value was less than a certain significance level were considered as detected.

### Symmetric difference of Robinson and Foulds

Symmetric difference of Robinson and Foulds was calculated with the program Treedist from the Phylip package [32]. First we obtained a non-zero distribution for the original data by calculating symmetric difference for each gene tree and consensus species tree. Then we calculated symmetrical difference for the gene trees with artificial transfers and normalized the obtained values by the average value of symmetric difference of the original data minus two standard deviations. For a signal generated through HGT to be considered significant we required that the normalized symmetric difference of the trees with artificial swaps and the species trees was more than zero.

SPR distance was calculated with the Efficient Evaluation of Edit Path (EEEP) algorithm [56].

### Analysis of bipartition spectra

For each gene family tree, 100 bootstrapped trees were generated and evaluated with the phyml program [31]. Bipartition tables were calculated from the individual trees using the program CONSENSE of the Phylip package [32]. We collected the highly supported bipartitions from each of the 236 families in one dataset by filtering out all bipartitions with bootstrap support values less than a given threshold (70 or 90%). The method of HGT detection consists in finding those bipartitions that exhibit a conflict with the consensus bipartition while having a high bootstrap support value.

*In silico *transfers were performed by flipping the two genomes in the original set of consensus bipartitions (see Table 1) and these bipartitions were then compared for compatibility with the bipartitions from the original gene families. Compatibility between bipartitions was calculated using in house PERL script.

## Authors' contributions

JPG conceived and directed the study, MSP wrote scripts and performed most of the reported analyses, JPG and MSP collaborated in writing the manuscript. Both authors read and approved the final manuscript

## Supplementary Material

Additional file 1 Power of HGT detection for *in-silico *gene exchanges and gene donations using the AU-test at different significance levels. Complementary tables for Figures 4 and 5. Click here for file

Additional file 2 Power of HGT detection using the symmetric difference of Robinson and Foulds distance. Complementary tables for Figure 6. Click here for file

Additional file 3 Power of HGT detection using bipartition spectra on genome data with *in silico *transfer. Complementary tables for Figure 8. Click here for file

## References

[B1] Gogarten JP, Doolittle WF, Lawrence JG (2002). Prokaryotic evolution in light of gene transfer. Mol Biol Evol.

[B2] Lawrence JG, Roth JR (1996). Selfish operons: horizontal transfer may drive the evolution of gene clusters. Genetics.

[B3] Ochman H, Lawrence JG, Groisman EA (2000). Lateral gene transfer and the nature of bacterial innovation. Nature.

[B4] Hacker J, Kaper JB (2000). Pathogenicity islands and the evolution of microbes. Annu Rev Microbiol.

[B5] Lawrence JG, Hendrickson H (2003). Lateral gene transfer: when will adolescence end?. Mol Microbiol.

[B6] Hsiao WW, Ung K, Aeschliman D, Bryan J, Finlay BB, Brinkman FS (2005). Evidence of a Large Novel Gene Pool Associated with Prokaryotic Genomic Islands. PLoS Genet.

[B7] Gogarten JP, Townsend JP (2005). Horizontal gene transfer, genome innovation and evolution. Nat Rev Microbiol.

[B8] Daubin V, Lerat E, Perriere G (2003). The source of laterally transferred genes in bacterial genomes. Genome Biol.

[B9] Thompson JR, Pacocha S, Pharino C, Klepac-Ceraj V, Hunt DE, Benoit J, Sarma-Rupavtarm R, Distel DL, Polz MF (2005). Genotypic diversity within a natural coastal bacterioplankton population. Science.

[B10] Omelchenko M, Makarova K, Wolf Y, Rogozin I, Koonin E (2003). Evolution of mosaic operons by horizontal gene transfer and gene displacement in situ. Genome Biology.

[B11] Huang J, Xu Y, Gogarten JP (2005). The Presence of a Haloarchaeal Type Tyrosyl-tRNA Synthetase Marks the Opisthokonts as Monophyletic. Mol Biol Evol.

[B12] Gophna U, Doolittle WF, Charlebois RL (2005). Weighted genome trees: refinements and applications. J Bacteriol.

[B13] Kurland CG, Canback B, Berg OG (2003). Horizontal gene transfer: a critical view. Proc Natl Acad Sci U S A.

[B14] Dutilh BE, Huynen MA, Bruno WJ, Snel B (2004). The consistent phylogenetic signal in genome trees revealed by reducing the impact of noise. J Mol Evol.

[B15] Daubin V, Ochman H (2004). Quartet Mapping and the Extent of Lateral Transfer in Bacterial Genomes. Mol Biol Evol.

[B16] Zhaxybayeva O, Gogarten JP (2003). An improved probability mapping approach to assess genome mosaicism. BMC Genomics.

[B17] Azad RK, Lawrence JG (2005). Use of Artificial Genomes in Assessing Methods for Atypical Gene Detection. PLoS Comput Biol.

[B18] Ragan MA (2001). On surrogate methods for detecting lateral gene transfer. FEMS Microbiol Lett.

[B19] Lawrence JG, Ochman H (2002). Reconciling the many faces of lateral gene transfer. Trends Microbiol.

[B20] Lawrence JG, Ochman H (1997). Amelioration of bacterial genomes: rates of change and exchange. J Mol Evol.

[B21] Koski LB, Morton RA, Golding GB (2001). Codon bias and base composition are poor indicators of horizontally transferred genes. Mol Biol Evol.

[B22] Wang B (2001). Limitations of compositional approach to identifying horizontally transferred genes. J Mol Evol.

[B23] Cortez DQ, Lazcano A, Becerra A (2005). Comparative analysis of methodologies for the detection of horizontally transferred genes: a reassessment of first-order Markov models. In Silico Biol.

[B24] Zhaxybayeva O, Lapierre P, Gogarten JP (2004). Genome mosaicism and organismal lineages. Trends Genet.

[B25] Lerat E, Daubin V, Moran NA (2003). From Gene Trees to Organismal Phylogeny in Prokaryotes:The Case of the gamma-Proteobacteria. PLoS Biol.

[B26] Shimodaira H (2002). An approximately unbiased test of phylogenetic tree selection. Syst Biol.

[B27] Robinson DR, Foulds LR (1981). Comparison of phylogenetic trees. Mathematical Biosciences.

[B28] Lento GM, Hickson RE, Chambers GK, Penny D (1995). Use of spectral analysis to test hypotheses on the origin of pinnipeds. Mol Biol Evol.

[B29] Shimodaira H, Hasegawa M (1999). Multiple Comparisons of Log-Likelihoods with Applications to Phylogenetic Inference. Mol Biol Evol.

[B30] Bapteste E, Boucher Y, Leigh J, Doolittle WF (2004). Phylogenetic reconstruction and lateral gene transfer. Trends in Microbiology.

[B31] Guindon S, Gascuel O (2003). A simple, fast, and accurate algorithm to estimate large phylogenies by maximum likelihood. Syst Biol.

[B32] Felsenstein J (1993). PHYLIP (Phylogeny Inference Package) version 3.6 Distributed by the author.. Department of Genetics, University of Washington, Seattle.

[B33] Bucknam J, Boucher Y, Bapteste E (2006). Refuting phylogenetic relationships. Biol Direct.

[B34] Ge F, Wang LS, Kim J (2005). The cobweb of life revealed by genome-scale estimates of horizontal gene transfer. PLoS Biol.

[B35] Goddard W KE (1994). The Agreement Metric for Labeled Binary Trees. Mathematical Biosciences.

[B36] Canback B, Tamas I, Andersson SG (2004). A phylogenomic study of endosymbiotic bacteria. Mol Biol Evol.

[B37] Gil R, Silva FJ, Zientz E, Delmotte F, Gonzalez-Candelas F, Latorre A, Rausell C, Kamerbeek J, Gadau J, Holldobler B, van Ham RC, Gross R, Moya A (2003). The genome sequence of Blochmannia floridanus: comparative analysis of reduced genomes. Proc Natl Acad Sci U S A.

[B38] Herbeck JT, Degnan PH, Wernegreen JJ (2005). Nonhomogeneous model of sequence evolution indicates independent origins of primary endosymbionts within the enterobacteriales (gamma-Proteobacteria). Mol Biol Evol.

[B39] Jain R, Rivera MC, Lake JA (1999). Horizontal gene transfer among genomes: the complexity hypothesis. Proc Natl Acad Sci U S A.

[B40] Koonin EV, Makarova KS, Aravind L (2001). Horizontal gene transfer in prokaryotes: quantification and classification. Annu Rev Microbiol.

[B41] Daubin V, Ochman H (2004). Bacterial Genomes as New Gene Homes: The Genealogy of ORFans in E. coli. Genome Res.

[B42] Lerat E, Daubin V, Ochman H, Moran NA (2005). Evolutionary Origins of Genomic Repertoires in Bacteria. PLoS Biol.

[B43] Ciccarelli FD, Doerks T, von Mering C, Creevey CJ, Snel B, Bork P (2006). Toward automatic reconstruction of a highly resolved tree of life. Science.

[B44] Ochman H, Lerat E, Daubin V (2005). Examining bacterial species under the specter of gene transfer and exchange. Proc Natl Acad Sci U S A.

[B45] Sokal RR, Freeman  (1995). Biometry: the principles and practice of statistics in biological research. 3rd ed.

[B46] Hillis DM, Bull JJ (1993). An empirical test of bootstrapping as a method for assessing confidence in phylogenetic analysis.. Syst Biol.

[B47] Zhaxybayeva O, Gogarten JP (2002). Bootstrap, Bayesian probability and maximum likelihood mapping: Exploring new tools for comparative genome analyses. BMC Genomics.

[B48] Huelsenbeck J, Rannala B (2004). Frequentist properties of Bayesian posterior probabilities of phylogenetic trees under simple and complex substitution models. Syst Biol.

[B49] Zhaxybayeva O, Gogarten JP, Charlebois RL, Doolittle WF, Papke RT (2006). Phylogenetic analyses of cyanobacterial genomes: Quantification of horizontal gene transfer events. Genome Res.

[B50] NCBI FTP server for Genome Assembly/Annotation Projects. ftp://ftp.ncbi.nlm.nih.gov/genomes/Bacteria/.

[B51] Altschul SF, Gish W, Miller W, Myers EW, Lipman DJ (1990). Basic local alignment search tool. J Mol Biol.

[B52] Montague MG, Hutchison CA (2000). Gene content phylogeny of herpesviruses. Proc Natl Acad Sci U S A.

[B53] Thompson JD, Higgins DG, Gibson TJ (1994). CLUSTAL W: improving the sensitivity of progressive multiple sequence alignment through sequence weighting, position-specific gap penalties and weight matrix choice.. Nucleic Acids Res.

[B54] Shimodaira H, Hasegawa M (2001). CONSEL: for assessing the confidence of phylogenetic tree selection. Bioinformatics.

[B55] Yang Z (1997). PAML: a program package for phylogenetic analysis by maximum likelihood. Comput Appl Biosci.

[B56] Beiko RG, Hamilton N (2006). Phylogenetic identification of lateral genetic transfer events. BMC Evol Biol.

