# Supplementary material for: The power of phylogenetic approaches to detect horizontally transferred genes
Source: BMC Evol Biol. 2007 Mar 21;7:45. doi: 10.1186/1471-2148-7-45 (PMC1847511; doi:10.1186/1471-2148-7-45)
Supplement: Additional file 1 — Power of HGT detection for in-silico gene exchanges and gene donations using the AU-test at different significance levels. Complementary tables for Figures 4 and 5. [file 1471-2148-7-45-S1.doc]

**Power of HGT detection for *in-silico* gene exchanges and gene donations using**

**the AU-test at different significance levels.**

**Complementary tables for Figures 4 and 5.**

**Table 1. Gene exchange between species.**

**Complementary tables for Figure 4.**

*Nu – is the number of families that showed significant conflict at a given significance level, % - relative number of families. Total number of families is 236.*

**A. Ordered by the position in the tree.**

| **AU Test: Gene Exchange Between Species**  *Blue – Significance level 0,05; Green – Significance level 0,01; values in %.* | | | | | | | | | | | | | | |
| --- | --- | --- | --- | --- | --- | --- | --- | --- | --- | --- | --- | --- | --- | --- |
|  |  | **5** | **7** | **8** | **1** | **12** | **13** | **6** | **2** | **4** | **3** | **11** | **9** | **10** |
| **/--------------** | **5** | 0 | 70 | 96 | 97 | 100 | 100 | 100 | 100 | 99 | 98 | 98 | 100 | 100 |
| **| /--------** | **7** | 51 | 0 | 88 | 89 | 98 | 98 | 97 | 98 | 94 | 95 | 100 | 100 | 100 |
| **| | ____/--** | **8** | 92 | 71 | 0 | 11 | 97 | 97 | 94 | 95 | 100 | 98 | 100 | 100 | 100 |
| **|_____| | \--** | **1** | 92 | 74 | 2 | 0 | 96 | 97 | 95 | 94 | 99 | 99 | 100 | 100 | 100 |
| **| | |_| __/--** | **12** | 99 | 96 | 92 | 92 | 0 | 11 | 98 | 98 | 100 | 100 | 100 | 100 | 100 |
| **| | |_/ \--** | **13** | 99 | 97 | 92 | 91 | 2 | 0 | 97 | 98 | 100 | 100 | 100 | 100 | 100 |
| **| | \__/--** | **6** | 98 | 94 | 83 | 82 | 96 | 95 | 0 | 11 | 100 | 100 | 98 | 100 | 100 |
| **| | \--** | **2** | 99 | 93 | 81 | 83 | 96 | 95 | 2 | 0 | 100 | 100 | 100 | 100 | 100 |
| **| |________/--** | **4** | 95 | 86 | 97 | 95 | 100 | 100 | 100 | 99 | 0 | 11 | 100 | 100 | 100 |
| **| \--** | **3** | 95 | 89 | 95 | 96 | 100 | 100 | 99 | 100 | 3 | 0 | 100 | 100 | 100 |
| **|__________/----** | **11** | 95 | 99 | 100 | 100 | 99 | 100 | 98 | 100 | 100 | 100 | 0 | 35 | 35 |
| **\_/--** | **9** | 99 | 100 | 99 | 99 | 99 | 100 | 100 | 100 | 100 | 99 | 14 | 0 | 11 |
| **\--** | **10** | 98 | 100 | 100 | 100 | 100 | 100 | 99 | 100 | 99 | 100 | 14 | 2 | 0 |

| **AU Test: Gene Exchange Between Species**  Blue – Significance level 10^-3^; Green – Significance level 10^-4^*; values in %.* | | | | | | | | | | | | | | |
| --- | --- | --- | --- | --- | --- | --- | --- | --- | --- | --- | --- | --- | --- | --- |
|  |  | **5** | **7** | **8** | **1** | **12** | **13** | **6** | **2** | **4** | **3** | **11** | **9** | **10** |
| **/--------------** | **5** | 0 | 32 | 80 | 75 | 96 | 97 | 94 | 94 | 91 | 90 | 86 | 91 | 91 |
| **| /--------** | **7** | 12 | 0 | 50 | 52 | 93 | 94 | 82 | 82 | 77 | 77 | 98 | 100 | 98 |
| **| | ____/--** | **8** | 54 | 28 | 0 | 2 | 80 | 79 | 65 | 66 | 90 | 89 | 99 | 97 | 99 |
| **|_____| | \--** | **1** | 58 | 31 | 0 | 0 | 79 | 82 | 66 | 63 | 90 | 88 | 99 | 98 | 99 |
| **| | |_| __/--** | **12** | 85 | 74 | 60 | 61 | 0 | 2 | 93 | 93 | 97 | 98 | 97 | 98 | 98 |
| **| | |_/ \--** | **13** | 89 | 78 | 59 | 59 | 0 | 0 | 92 | 93 | 99 | 99 | 99 | 98 | 99 |
| **| | \__/--** | **6** | 78 | 61 | 44 | 43 | 85 | 84 | 0 | 2 | 97 | 97 | 97 | 99 | 97 |
| **| | \--** | **2** | 77 | 63 | 42 | 40 | 86 | 86 | 0 | 0 | 97 | 97 | 98 | 98 | 98 |
| **| |________/--** | **4** | 77 | 59 | 69 | 72 | 92 | 93 | 92 | 87 | 0 | 2 | 98 | 99 | 98 |
| **| \--** | **3** | 73 | 59 | 72 | 70 | 92 | 92 | 90 | 90 | 1 | 0 | 98 | 99 | 97 |
| **|__________/----** | **11** | 72 | 92 | 90 | 88 | 89 | 90 | 92 | 94 | 93 | 91 | 0 | 4 | 4 |
| **\_/--** | **9** | 75 | 92 | 93 | 94 | 93 | 87 | 94 | 93 | 92 | 93 | 2 | 0 | 2 |
| **\--** | **10** | 78 | 89 | 96 | 94 | 92 | 96 | 92 | 92 | 94 | 92 | 1 | 1 | 0 |

**B. Ordered by species’ numbers.**

| **flips** | **significance level** | |  | **significance level** | |  | **significance level** | |  | **significance level** | |
| --- | --- | --- | --- | --- | --- | --- | --- | --- | --- | --- | --- |
|  | **0,05** | |  | **e-2** | |  | **e-3** | |  | **e-4** | |
|  | **Nu** | **%** |  | **Nu** | **%** |  | **Nu** | **%** |  | **Nu** | **%** |
| 1-2 | 223 | 94 |  | 195 | 83 |  | 149 | 63 |  | 95 | 40 |
| 1-3 | 233 | 99 |  | 226 | 96 |  | 208 | 88 |  | 165 | 70 |
| 1-4 | 234 | 99 |  | 225 | 95 |  | 212 | 90 |  | 171 | 72 |
| 1-5 | 228 | 97 |  | 217 | 92 |  | 178 | 75 |  | 137 | 58 |
| 1-6 | 224 | 95 |  | 194 | 82 |  | 155 | 66 |  | 102 | 43 |
| 1-7 | 209 | 89 |  | 175 | 74 |  | 122 | 52 |  | 72 | 31 |
| 1-8 | 26 | 11 |  | 4 | 2 |  | 4 | 2 |  | 1 | 0 |
| 1-9 | 236 | 100 |  | 234 | 99 |  | 231 | 98 |  | 222 | 94 |
| 1-10 | 236 | 100 |  | 236 | 100 |  | 234 | 99 |  | 223 | 94 |
| 1-11 | 236 | 100 |  | 235 | 100 |  | 233 | 99 |  | 208 | 88 |
| 1-12 | 227 | 96 |  | 216 | 92 |  | 187 | 79 |  | 145 | 61 |
| 1-13 | 228 | 97 |  | 215 | 91 |  | 193 | 82 |  | 140 | 59 |
| 2-3 | 235 | 100 |  | 235 | 100 |  | 230 | 97 |  | 213 | 90 |
| 2-4 | 235 | 100 |  | 233 | 99 |  | 230 | 97 |  | 205 | 87 |
| 2-5 | 235 | 100 |  | 233 | 99 |  | 222 | 94 |  | 182 | 77 |
| 2-6 | 26 | 11 |  | 5 | 2 |  | 4 | 2 |  | 1 | 0 |
| 2-7 | 231 | 98 |  | 220 | 93 |  | 194 | 82 |  | 148 | 63 |
| 2-8 | 224 | 95 |  | 191 | 81 |  | 156 | 66 |  | 100 | 42 |
| 2-9 | 236 | 100 |  | 235 | 100 |  | 232 | 98 |  | 219 | 93 |
| 2-10 | 236 | 100 |  | 236 | 100 |  | 231 | 98 |  | 216 | 92 |
| 2-11 | 236 | 100 |  | 236 | 100 |  | 232 | 98 |  | 222 | 94 |
| 2-12 | 231 | 98 |  | 226 | 96 |  | 219 | 93 |  | 204 | 86 |
| 2-13 | 232 | 98 |  | 225 | 95 |  | 220 | 93 |  | 202 | 86 |
| 3-4 | 26 | 11 |  | 6 | 3 |  | 4 | 2 |  | 2 | 1 |
| 3-5 | 232 | 98 |  | 225 | 95 |  | 212 | 90 |  | 172 | 73 |
| 3-6 | 235 | 100 |  | 233 | 99 |  | 228 | 97 |  | 212 | 90 |
| 3-7 | 224 | 95 |  | 209 | 89 |  | 181 | 77 |  | 140 | 59 |
| 3-8 | 232 | 98 |  | 224 | 95 |  | 210 | 89 |  | 170 | 72 |
| 3-9 | 236 | 100 |  | 234 | 99 |  | 233 | 99 |  | 219 | 93 |
| 3-10 | 235 | 100 |  | 235 | 100 |  | 230 | 97 |  | 218 | 92 |
| 3-11 | 236 | 100 |  | 235 | 100 |  | 232 | 98 |  | 215 | 91 |
| 3-12 | 235 | 100 |  | 235 | 100 |  | 232 | 98 |  | 217 | 92 |
| 3-13 | 235 | 100 |  | 235 | 100 |  | 233 | 99 |  | 217 | 92 |
| 4-5 | 234 | 99 |  | 225 | 95 |  | 214 | 91 |  | 181 | 77 |
| 4-6 | 235 | 100 |  | 235 | 100 |  | 229 | 97 |  | 216 | 92 |
| 4-7 | 223 | 94 |  | 204 | 86 |  | 181 | 77 |  | 139 | 59 |
| 4-8 | 235 | 100 |  | 228 | 97 |  | 212 | 90 |  | 164 | 69 |
| 4-9 | 236 | 100 |  | 236 | 100 |  | 234 | 99 |  | 217 | 92 |
| 4-10 | 235 | 100 |  | 233 | 99 |  | 232 | 98 |  | 222 | 94 |
| 4-11 | 236 | 100 |  | 235 | 100 |  | 232 | 98 |  | 219 | 93 |
| 4-12 | 235 | 100 |  | 235 | 100 |  | 230 | 97 |  | 217 | 92 |
| 4-13 | 235 | 100 |  | 235 | 100 |  | 234 | 99 |  | 219 | 93 |
| 5-6 | 235 | 100 |  | 231 | 98 |  | 221 | 94 |  | 184 | 78 |
| 5-7 | 166 | 70 |  | 120 | 51 |  | 76 | 32 |  | 29 | 12 |
| 5-8 | 227 | 96 |  | 216 | 92 |  | 188 | 80 |  | 128 | 54 |
| 5-9 | 236 | 100 |  | 234 | 99 |  | 214 | 91 |  | 177 | 75 |
| 5-10 | 235 | 100 |  | 232 | 98 |  | 214 | 91 |  | 185 | 78 |
| 5-11 | 232 | 98 |  | 224 | 95 |  | 204 | 86 |  | 169 | 72 |
| 5-12 | 235 | 100 |  | 234 | 99 |  | 227 | 96 |  | 201 | 85 |
| 5-13 | 235 | 100 |  | 234 | 99 |  | 230 | 97 |  | 209 | 89 |
| 6-7 | 230 | 97 |  | 221 | 94 |  | 194 | 82 |  | 143 | 61 |
| 6-8 | 223 | 94 |  | 195 | 83 |  | 154 | 65 |  | 104 | 44 |
| 6-9 | 236 | 100 |  | 235 | 100 |  | 234 | 99 |  | 221 | 94 |
| 6-10 | 235 | 100 |  | 234 | 99 |  | 230 | 97 |  | 216 | 92 |
| 6-11 | 232 | 98 |  | 232 | 98 |  | 228 | 97 |  | 218 | 92 |
| 6-12 | 232 | 98 |  | 226 | 96 |  | 219 | 93 |  | 200 | 85 |
| 6-13 | 229 | 97 |  | 225 | 95 |  | 217 | 92 |  | 198 | 84 |
| 7-8 | 207 | 88 |  | 167 | 71 |  | 117 | 50 |  | 66 | 28 |
| 7-9 | 236 | 100 |  | 236 | 100 |  | 235 | 100 |  | 217 | 92 |
| 7-10 | 236 | 100 |  | 236 | 100 |  | 231 | 98 |  | 209 | 89 |
| 7-11 | 235 | 100 |  | 234 | 99 |  | 232 | 98 |  | 216 | 92 |
| 7-12 | 231 | 98 |  | 227 | 96 |  | 219 | 93 |  | 175 | 74 |
| 7-13 | 232 | 98 |  | 229 | 97 |  | 221 | 94 |  | 185 | 78 |
| 8-9 | 235 | 100 |  | 234 | 99 |  | 230 | 97 |  | 220 | 93 |
| 8-10 | 236 | 100 |  | 236 | 100 |  | 234 | 99 |  | 227 | 96 |
| 8-11 | 236 | 100 |  | 235 | 100 |  | 233 | 99 |  | 213 | 90 |
| 8-12 | 229 | 97 |  | 216 | 92 |  | 189 | 80 |  | 141 | 60 |
| 8-13 | 230 | 97 |  | 216 | 92 |  | 187 | 79 |  | 139 | 59 |
| 9-10 | 25 | 11 |  | 4 | 2 |  | 4 | 2 |  | 2 | 1 |
| 9-11 | 83 | 35 |  | 32 | 14 |  | 9 | 4 |  | 5 | 2 |
| 9-12 | 236 | 100 |  | 233 | 99 |  | 231 | 98 |  | 220 | 93 |
| 9-13 | 236 | 100 |  | 236 | 100 |  | 232 | 98 |  | 206 | 87 |
| 10-11 | 83 | 35 |  | 34 | 14 |  | 9 | 4 |  | 2 | 1 |
| 10-12 | 235 | 100 |  | 235 | 100 |  | 231 | 98 |  | 217 | 92 |
| 10-13 | 236 | 100 |  | 236 | 100 |  | 234 | 99 |  | 226 | 96 |
| 11-12 | 236 | 100 |  | 233 | 99 |  | 230 | 97 |  | 211 | 89 |
| 11-13 | 236 | 100 |  | 235 | 100 |  | 233 | 99 |  | 213 | 90 |
| 12-13 | 25 | 11 |  | 5 | 2 |  | 4 | 2 |  | 1 | 0 |

**Table 2. AU Test . Gene donation with replacement.**

**Complementary tables for Figure 5.**

*Nu – is the number of families that showed significant conflict at a given significance level, % - relative number of families. Total number of families is 236.*

**A. Ordered by the position in the tree**

| **AU Test: Gene Donation with Replacement**  *Significance level 0,05; values in %.* | | | | | | | | | | | | | | |
| --- | --- | --- | --- | --- | --- | --- | --- | --- | --- | --- | --- | --- | --- | --- |
|  |  | **5** | **7** | **8** | **1** | **12** | **13** | **6** | **2** | **4** | **3** | **11** | **9** | **10** |
| **/--------------** | **5** | 0 | 87 | 100 | 100 | 100 | 100 | 100 | 100 | 100 | 100 | 88 | 100 | 100 |
| **| /--------** | **7** | 75 | 0 | 100 | 100 | 100 | 100 | 100 | 100 | 98 | 99 | 98 | 100 | 100 |
| **| | ____/--** | **8** | 100 | 100 | 0 | 9 | 100 | 100 | 100 | 100 | 100 | 100 | 100 | 100 | 100 |
| **|_____| | \--** | **1** | 100 | 100 | 11 | 0 | 100 | 100 | 100 | 100 | 100 | 100 | 100 | 100 | 100 |
| **| | |_| __/--** | **12** | 99 | 97 | 86 | 88 | 0 | 11 | 86 | 85 | 97 | 97 | 100 | 100 | 100 |
| **| | |_/ \--** | **13** | 99 | 96 | 88 | 89 | 11 | 0 | 87 | 86 | 97 | 97 | 100 | 100 | 100 |
| **| | \__/--** | **6** | 99 | 96 | 89 | 90 | 86 | 86 | 0 | 11 | 98 | 97 | 100 | 99 | 100 |
| **| | \--** | **2** | 99 | 96 | 85 | 87 | 84 | 84 | 12 | 0 | 97 | 97 | 100 | 100 | 100 |
| **| |________/--** | **4** | 98 | 94 | 98 | 97 | 99 | 100 | 100 | 99 | 0 | 11 | 100 | 100 | 100 |
| **| \--** | **3** | 98 | 94 | 99 | 98 | 100 | 99 | 99 | 100 | 10 | 0 | 99 | 100 | 100 |
| **|__________/----** | **11** | 76 | 100 | 100 | 100 | 100 | 100 | 100 | 100 | 100 | 100 | 0 | 80 | 80 |
| **\_/--** | **9** | 64 | 100 | 100 | 100 | 99 | 100 | 100 | 100 | 100 | 100 | 25 | 0 | 11 |
| **\--** | **10** | 66 | 100 | 100 | 100 | 100 | 100 | 100 | 100 | 100 | 100 | 25 | 11 | 0 |

| **AU Test: Gene Donation with Replacement**  *Significance level 0,01; values in %.* | | | | | | | | | | | | | | |
| --- | --- | --- | --- | --- | --- | --- | --- | --- | --- | --- | --- | --- | --- | --- |
|  |  | **5** | **7** | **8** | **1** | **12** | **13** | **6** | **2** | **4** | **3** | **11** | **9** | **10** |
| **/--------------** | **5** | 0 | 73 | 100 | 100 | 99 | 100 | 99 | 100 | 100 | 100 | 69 | 100 | 100 |
| **| /--------** | **7** | 47 | 0 | 98 | 99 | 100 | 100 | 99 | 100 | 94 | 94 | 91 | 100 | 100 |
| **| | ____/--** | **8** | 100 | 100 | 0 | 4 | 99 | 100 | 100 | 99 | 100 | 100 | 99 | 99 | 99 |
| **|_____| | \--** | **1** | 99 | 100 | 3 | 0 | 100 | 100 | 100 | 100 | 100 | 99 | 99 | 99 | 99 |
| **| | |_| __/--** | **12** | 97 | 90 | 76 | 75 | 0 | 3 | 69 | 69 | 92 | 92 | 99 | 100 | 100 |
| **| | |_/ \--** | **13** | 97 | 90 | 75 | 76 | 2 | 0 | 70 | 69 | 93 | 92 | 99 | 100 | 100 |
| **| | \__/--** | **6** | 97 | 89 | 69 | 73 | 67 | 66 | 0 | 3 | 90 | 92 | 99 | 99 | 100 |
| **| | \--** | **2** | 97 | 85 | 68 | 69 | 64 | 64 | 3 | 0 | 89 | 89 | 100 | 100 | 100 |
| **| |________/--** | **4** | 95 | 84 | 95 | 94 | 99 | 98 | 100 | 99 | 0 | 3 | 98 | 99 | 100 |
| **| \--** | **3** | 95 | 91 | 96 | 97 | 99 | 98 | 99 | 98 | 3 | 0 | 98 | 99 | 100 |
| **|__________/----** | **11** | 52 | 100 | 100 | 100 | 100 | 99 | 100 | 100 | 100 | 100 | 0 | 61 | 62 |
| **\_/--** | **9** | 37 | 100 | 100 | 100 | 99 | 99 | 100 | 100 | 100 | 100 | 6 | 0 | 4 |
| **\--** | **10** | 40 | 99 | 99 | 100 | 100 | 99 | 99 | 100 | 100 | 100 | 8 | 3 | 0 |

| **AU Test: Gene Donation with Replacement**  *Significance level 10^-3^; values in %.* | | | | | | | | | | | | | | |
| --- | --- | --- | --- | --- | --- | --- | --- | --- | --- | --- | --- | --- | --- | --- |
|  |  | **5** | **7** | **8** | **1** | **12** | **13** | **6** | **2** | **4** | **3** | **11** | **9** | **10** |
| **/--------------** | **5** | 0 | 57 | 100 | 99 | 97 | 98 | 97 | 99 | 97 | 98 | 51 | 97 | 99 |
| **| /--------** | **7** | 31 | 0 | 94 | 95 | 99 | 99 | 98 | 100 | 88 | 89 | 80 | 98 | 99 |
| **| | ____/--** | **8** | 98 | 98 | 0 | 2 | 97 | 97 | 97 | 97 | 99 | 99 | 98 | 97 | 98 |
| **|_____| | \--** | **1** | 97 | 98 | 3 | 0 | 99 | 98 | 99 | 98 | 96 | 98 | 97 | 97 | 98 |
| **| | |_| __/--** | **12** | 92 | 75 | 50 | 55 | 0 | 2 | 47 | 44 | 82 | 84 | 95 | 99 | 98 |
| **| | |_/ \--** | **13** | 93 | 75 | 53 | 57 | 2 | 0 | 48 | 44 | 83 | 85 | 95 | 98 | 99 |
| **| | \__/--** | **6** | 88 | 73 | 54 | 55 | 50 | 50 | 0 | 2 | 78 | 80 | 95 | 97 | 98 |
| **| | \--** | **2** | 86 | 72 | 50 | 53 | 50 | 48 | 2 | 0 | 79 | 76 | 97 | 98 | 98 |
| **| |________/--** | **4** | 86 | 70 | 90 | 91 | 98 | 97 | 97 | 95 | 0 | 2 | 95 | 98 | 98 |
| **| \--** | **3** | 89 | 76 | 91 | 92 | 98 | 97 | 99 | 97 | 3 | 0 | 96 | 98 | 98 |
| **|__________/----** | **11** | 30 | 97 | 99 | 97 | 97 | 99 | 99 | 98 | 98 | 97 | 0 | 42 | 41 |
| **\_/--** | **9** | 20 | 96 | 98 | 98 | 96 | 98 | 98 | 98 | 97 | 99 | 2 | 0 | 3 |
| **\--** | **10** | 19 | 94 | 98 | 98 | 98 | 97 | 98 | 97 | 98 | 96 | 2 | 2 | 0 |

| **AU Test: Gene Donation with Replacement**  *Significance level 10^-4^; values in %.* | | | | | | | | | | | | | | |
| --- | --- | --- | --- | --- | --- | --- | --- | --- | --- | --- | --- | --- | --- | --- |
|  |  | **5** | **7** | **8** | **1** | **12** | **13** | **6** | **2** | **4** | **3** | **11** | **9** | **10** |
| **/--------------** | **5** | 0 | 33 | 93 | 94 | 91 | 93 | 90 | 95 | 93 | 89 | 30 | 83 | 89 |
| **| /--------** | **7** | 19 | 0 | 86 | 81 | 94 | 92 | 91 | 97 | 73 | 75 | 64 | 93 | 91 |
| **| | ____/--** | **8** | 89 | 94 | 0 | 1 | 91 | 92 | 92 | 93 | 95 | 95 | 94 | 88 | 93 |
| **|_____| | \--** | **1** | 92 | 93 | 1 | 0 | 94 | 93 | 91 | 92 | 87 | 92 | 92 | 91 | 92 |
| **| | |_| __/--** | **12** | 70 | 54 | 30 | 29 | 0 | 1 | 26 | 25 | 62 | 64 | 87 | 92 | 92 |
| **| | |_/ \--** | **13** | 78 | 56 | 29 | 30 | 1 | 0 | 27 | 29 | 62 | 62 | 89 | 89 | 97 |
| **| | \__/--** | **6** | 70 | 58 | 36 | 38 | 29 | 28 | 0 | 1 | 56 | 59 | 84 | 90 | 93 |
| **| | \--** | **2** | 67 | 56 | 32 | 37 | 32 | 31 | 1 | 0 | 57 | 58 | 87 | 90 | 94 |
| **| |________/--** | **4** | 72 | 53 | 80 | 76 | 92 | 92 | 92 | 89 | 0 | 1 | 84 | 95 | 93 |
| **| \--** | **3** | 76 | 58 | 81 | 80 | 93 | 90 | 92 | 89 | 1 | 0 | 85 | 93 | 90 |
| **|__________/----** | **11** | 18 | 84 | 90 | 91 | 91 | 94 | 95 | 93 | 94 | 93 | 0 | 21 | 20 |
| **\_/--** | **9** | 10 | 84 | 93 | 94 | 91 | 94 | 93 | 92 | 89 | 95 | 1 | 0 | 2 |
| **\--** | **10** | 10 | 82 | 89 | 92 | 90 | 91 | 92 | 92 | 91 | 89 | 1 | 0 | 0 |

**B. Ordered by species’ numbers.**

| **donate** | **significance level** | |  | **significance level** | |  | **significance level** | | |  | | **significance level** | |
| --- | --- | --- | --- | --- | --- | --- | --- | --- | --- | --- | --- | --- | --- |
|  | **0,05** | |  | **e-2** | |  | **e-3** | | |  | | **e-4** | |
|  | **Nu** | **%** |  | **Nu** | **%** |  | **Nu** | **%** | |  | | **Nu** | **%** |
| 1-2 | 235 | 100 |  | 235 | 100 |  | 231 | 98 |  | | 216 | | 92 |
| 1-3 | 235 | 100 |  | 234 | 99 |  | 232 | 98 |  | | 216 | | 92 |
| 1-4 | 236 | 100 |  | 235 | 100 |  | 227 | 96 |  | | 205 | | 87 |
| 1-5 | 235 | 100 |  | 234 | 99 |  | 229 | 97 |  | | 217 | | 92 |
| 1-6 | 236 | 100 |  | 235 | 100 |  | 234 | 99 |  | | 214 | | 91 |
| 1-7 | 236 | 100 |  | 235 | 100 |  | 231 | 98 |  | | 220 | | 93 |
| 1-8 | 27 | 11 |  | 7 | 3 |  | 6 | 3 |  | | 2 | | 1 |
| 1-9 | 235 | 100 |  | 234 | 99 |  | 230 | 97 |  | | 214 | | 91 |
| 1-10 | 236 | 100 |  | 234 | 99 |  | 231 | 98 |  | | 218 | | 92 |
| 1-11 | 235 | 100 |  | 233 | 99 |  | 229 | 97 |  | | 217 | | 92 |
| 1-12 | 236 | 100 |  | 236 | 100 |  | 233 | 99 |  | | 221 | | 94 |
| 1-13 | 236 | 100 |  | 236 | 100 |  | 232 | 98 |  | | 220 | | 93 |
| 2-1 | 206 | 87 |  | 164 | 69 |  | 126 | 53 |  | | 88 | | 37 |
| 2-3 | 229 | 97 |  | 210 | 89 |  | 180 | 76 |  | | 137 | | 58 |
| 2-4 | 230 | 97 |  | 209 | 89 |  | 186 | 79 |  | | 134 | | 57 |
| 2-5 | 234 | 99 |  | 228 | 97 |  | 202 | 86 |  | | 159 | | 67 |
| 2-6 | 29 | 12 |  | 8 | 3 |  | 5 | 2 |  | | 3 | | 1 |
| 2-7 | 226 | 96 |  | 201 | 85 |  | 171 | 72 |  | | 132 | | 56 |
| 2-8 | 200 | 85 |  | 161 | 68 |  | 119 | 50 |  | | 75 | | 32 |
| 2-9 | 235 | 100 |  | 235 | 100 |  | 232 | 98 |  | | 213 | | 90 |
| 2-10 | 235 | 100 |  | 235 | 100 |  | 232 | 98 |  | | 221 | | 94 |
| 2-11 | 236 | 100 |  | 235 | 100 |  | 228 | 97 |  | | 206 | | 87 |
| 2-12 | 198 | 84 |  | 152 | 64 |  | 117 | 50 |  | | 76 | | 32 |
| 2-13 | 198 | 84 |  | 150 | 64 |  | 114 | 48 |  | | 73 | | 31 |
| 3-1 | 231 | 98 |  | 228 | 97 |  | 218 | 92 |  | | 189 | | 80 |
| 3-2 | 235 | 100 |  | 232 | 98 |  | 229 | 97 |  | | 210 | | 89 |
| 3-4 | 24 | 10 |  | 8 | 3 |  | 6 | 3 |  | | 3 | | 1 |
| 3-5 | 232 | 98 |  | 225 | 95 |  | 211 | 89 |  | | 180 | | 76 |
| 3-6 | 234 | 99 |  | 234 | 99 |  | 233 | 99 |  | | 217 | | 92 |
| 3-7 | 223 | 94 |  | 214 | 91 |  | 179 | 76 |  | | 138 | | 58 |
| 3-8 | 233 | 99 |  | 227 | 96 |  | 214 | 91 |  | | 190 | | 81 |
| 3-9 | 236 | 100 |  | 234 | 99 |  | 231 | 98 |  | | 219 | | 93 |
| 3-10 | 236 | 100 |  | 235 | 100 |  | 231 | 98 |  | | 212 | | 90 |
| 3-11 | 234 | 99 |  | 232 | 98 |  | 226 | 96 |  | | 200 | | 85 |
| 3-12 | 235 | 100 |  | 234 | 99 |  | 231 | 98 |  | | 219 | | 93 |
| 3-13 | 234 | 99 |  | 232 | 98 |  | 229 | 97 |  | | 213 | | 90 |
| 4-1 | 230 | 97 |  | 223 | 94 |  | 215 | 91 |  | | 180 | | 76 |
| 4-2 | 234 | 99 |  | 233 | 99 |  | 224 | 95 |  | | 211 | | 89 |
| 4-3 | 26 | 11 |  | 6 | 3 |  | 5 | 2 |  | | 3 | | 1 |
| 4-5 | 232 | 98 |  | 224 | 95 |  | 202 | 86 |  | | 169 | | 72 |
| 4-6 | 235 | 100 |  | 235 | 100 |  | 229 | 97 |  | | 216 | | 92 |
| 4-7 | 221 | 94 |  | 199 | 84 |  | 166 | 70 |  | | 126 | | 53 |
| 4-8 | 231 | 98 |  | 225 | 95 |  | 213 | 90 |  | | 189 | | 80 |
| 4-9 | 235 | 100 |  | 233 | 99 |  | 232 | 98 |  | | 225 | | 95 |
| 4-10 | 236 | 100 |  | 235 | 100 |  | 232 | 98 |  | | 220 | | 93 |
| 4-11 | 235 | 100 |  | 232 | 98 |  | 224 | 95 |  | | 199 | | 84 |
| 4-12 | 234 | 99 |  | 233 | 99 |  | 231 | 98 |  | | 217 | | 92 |
| 4-13 | 235 | 100 |  | 232 | 98 |  | 228 | 97 |  | | 216 | | 92 |
| 5-1 | 236 | 100 |  | 236 | 100 |  | 234 | 99 |  | | 223 | | 94 |
| 5-2 | 236 | 100 |  | 235 | 100 |  | 233 | 99 |  | | 224 | | 95 |
| 5-3 | 236 | 100 |  | 236 | 100 |  | 231 | 98 |  | | 209 | | 89 |
| 5-4 | 236 | 100 |  | 236 | 100 |  | 228 | 97 |  | | 220 | | 93 |
| 5-6 | 235 | 100 |  | 233 | 99 |  | 228 | 97 |  | | 213 | | 90 |
| 5-7 | 205 | 87 |  | 173 | 73 |  | 135 | 57 |  | | 78 | | 33 |
| 5-8 | 236 | 100 |  | 236 | 100 |  | 235 | 100 |  | | 219 | | 93 |
| 5-9 | 236 | 100 |  | 235 | 100 |  | 228 | 97 |  | | 197 | | 83 |
| 5-10 | 236 | 100 |  | 235 | 100 |  | 233 | 99 |  | | 209 | | 89 |
| 5-11 | 208 | 88 |  | 162 | 69 |  | 120 | 51 |  | | 71 | | 30 |
| 5-12 | 236 | 100 |  | 233 | 99 |  | 229 | 97 |  | | 214 | | 91 |
| 5-13 | 236 | 100 |  | 235 | 100 |  | 231 | 98 |  | | 219 | | 93 |
| 6-1 | 212 | 90 |  | 172 | 73 |  | 129 | 55 |  | | 89 | | 38 |
| 6-2 | 25 | 11 |  | 7 | 3 |  | 5 | 2 |  | | 2 | | 1 |
| 6-3 | 229 | 97 |  | 216 | 92 |  | 188 | 80 |  | | 140 | | 59 |
| 6-4 | 232 | 98 |  | 212 | 90 |  | 183 | 78 |  | | 133 | | 56 |
| 6-5 | 234 | 99 |  | 229 | 97 |  | 208 | 88 |  | | 165 | | 70 |
| 6-7 | 227 | 96 |  | 209 | 89 |  | 172 | 73 |  | | 138 | | 58 |
| 6-8 | 210 | 89 |  | 162 | 69 |  | 128 | 54 |  | | 84 | | 36 |
| 6-9 | 234 | 99 |  | 233 | 99 |  | 229 | 97 |  | | 213 | | 90 |
| 6-10 | 236 | 100 |  | 236 | 100 |  | 232 | 98 |  | | 220 | | 93 |
| 6-11 | 235 | 100 |  | 234 | 99 |  | 225 | 95 |  | | 199 | | 84 |
| 6-12 | 204 | 86 |  | 159 | 67 |  | 117 | 50 |  | | 69 | | 29 |
| 6-13 | 204 | 86 |  | 156 | 66 |  | 118 | 50 |  | | 67 | | 28 |
| 7-1 | 235 | 100 |  | 233 | 99 |  | 224 | 95 |  | | 191 | | 81 |
| 7-2 | 235 | 100 |  | 235 | 100 |  | 235 | 100 |  | | 229 | | 97 |
| 7-3 | 233 | 99 |  | 222 | 94 |  | 209 | 89 |  | | 178 | | 75 |
| 7-4 | 232 | 98 |  | 221 | 94 |  | 208 | 88 |  | | 173 | | 73 |
| 7-5 | 176 | 75 |  | 111 | 47 |  | 72 | 31 |  | | 45 | | 19 |
| 7-6 | 236 | 100 |  | 234 | 99 |  | 231 | 98 |  | | 214 | | 91 |
| 7-8 | 236 | 100 |  | 231 | 98 |  | 223 | 94 |  | | 203 | | 86 |
| 7-9 | 236 | 100 |  | 235 | 100 |  | 231 | 98 |  | | 219 | | 93 |
| 7-10 | 236 | 100 |  | 235 | 100 |  | 233 | 99 |  | | 214 | | 91 |
| 7-11 | 232 | 98 |  | 215 | 91 |  | 189 | 80 |  | | 150 | | 64 |
| 7-12 | 236 | 100 |  | 236 | 100 |  | 233 | 99 |  | | 222 | | 94 |
| 7-13 | 236 | 100 |  | 236 | 100 |  | 233 | 99 |  | | 218 | | 92 |
| 8-1 | 22 | 9 |  | 9 | 4 |  | 5 | 2 |  | | 3 | | 1 |
| 8-2 | 235 | 100 |  | 234 | 99 |  | 230 | 97 |  | | 219 | | 93 |
| 8-3 | 236 | 100 |  | 236 | 100 |  | 234 | 99 |  | | 225 | | 95 |
| 8-4 | 236 | 100 |  | 236 | 100 |  | 234 | 99 |  | | 224 | | 95 |
| 8-5 | 236 | 100 |  | 236 | 100 |  | 232 | 98 |  | | 210 | | 89 |
| 8-6 | 236 | 100 |  | 235 | 100 |  | 229 | 97 |  | | 216 | | 92 |
| 8-7 | 236 | 100 |  | 236 | 100 |  | 231 | 98 |  | | 221 | | 94 |
| 8-9 | 235 | 100 |  | 233 | 99 |  | 229 | 97 |  | | 208 | | 88 |
| 8-10 | 236 | 100 |  | 234 | 99 |  | 232 | 98 |  | | 219 | | 93 |
| 8-11 | 236 | 100 |  | 234 | 99 |  | 231 | 98 |  | | 223 | | 94 |
| 8-12 | 235 | 100 |  | 234 | 99 |  | 230 | 97 |  | | 215 | | 91 |
| 8-13 | 235 | 100 |  | 235 | 100 |  | 228 | 97 |  | | 217 | | 92 |
| 9-1 | 236 | 100 |  | 236 | 100 |  | 232 | 98 |  | | 223 | | 94 |
| 9-2 | 235 | 100 |  | 235 | 100 |  | 231 | 98 |  | | 216 | | 92 |
| 9-3 | 236 | 100 |  | 236 | 100 |  | 234 | 99 |  | | 224 | | 95 |
| 9-4 | 236 | 100 |  | 235 | 100 |  | 228 | 97 |  | | 210 | | 89 |
| 9-5 | 151 | 64 |  | 88 | 37 |  | 48 | 20 |  | | 23 | | 10 |
| 9-6 | 236 | 100 |  | 236 | 100 |  | 231 | 98 |  | | 220 | | 93 |
| 9-7 | 236 | 100 |  | 235 | 100 |  | 226 | 96 |  | | 198 | | 84 |
| 9-8 | 236 | 100 |  | 235 | 100 |  | 231 | 98 |  | | 219 | | 93 |
| 9-10 | 27 | 11 |  | 9 | 4 |  | 7 | 3 |  | | 4 | | 2 |
| 9-11 | 58 | 25 |  | 14 | 6 |  | 5 | 2 |  | | 2 | | 1 |
| 9-12 | 234 | 99 |  | 233 | 99 |  | 227 | 96 |  | | 215 | | 91 |
| 9-13 | 236 | 100 |  | 234 | 99 |  | 232 | 98 |  | | 221 | | 94 |
| 10-1 | 236 | 100 |  | 235 | 100 |  | 232 | 98 |  | | 217 | | 92 |
| 10-2 | 236 | 100 |  | 235 | 100 |  | 230 | 97 |  | | 216 | | 92 |
| 10-3 | 236 | 100 |  | 236 | 100 |  | 227 | 96 |  | | 211 | | 89 |
| 10-4 | 236 | 100 |  | 235 | 100 |  | 231 | 98 |  | | 215 | | 91 |
| 10-5 | 155 | 66 |  | 95 | 40 |  | 46 | 19 |  | | 24 | | 10 |
| 10-6 | 236 | 100 |  | 234 | 99 |  | 232 | 98 |  | | 217 | | 92 |
| 10-7 | 235 | 100 |  | 234 | 99 |  | 223 | 94 |  | | 194 | | 82 |
| 10-8 | 236 | 100 |  | 234 | 99 |  | 231 | 98 |  | | 211 | | 89 |
| 10-9 | 26 | 11 |  | 7 | 3 |  | 5 | 2 |  | | 1 | | 0 |
| 10-11 | 58 | 25 |  | 20 | 8 |  | 5 | 2 |  | | 3 | | 1 |
| 10-12 | 236 | 100 |  | 235 | 100 |  | 231 | 98 |  | | 213 | | 90 |
| 10-13 | 235 | 100 |  | 234 | 99 |  | 230 | 97 |  | | 214 | | 91 |
| 11-1 | 236 | 100 |  | 236 | 100 |  | 228 | 97 |  | | 214 | | 91 |
| 11-2 | 236 | 100 |  | 236 | 100 |  | 231 | 98 |  | | 220 | | 93 |
| 11-3 | 236 | 100 |  | 236 | 100 |  | 230 | 97 |  | | 219 | | 93 |
| 11-4 | 236 | 100 |  | 235 | 100 |  | 232 | 98 |  | | 221 | | 94 |
| 11-5 | 179 | 76 |  | 122 | 52 |  | 70 | 30 |  | | 42 | | 18 |
| 11-6 | 236 | 100 |  | 235 | 100 |  | 234 | 99 |  | | 225 | | 95 |
| 11-7 | 235 | 100 |  | 235 | 100 |  | 228 | 97 |  | | 198 | | 84 |
| 11-8 | 235 | 100 |  | 235 | 100 |  | 233 | 99 |  | | 213 | | 90 |
| 11-9 | 189 | 80 |  | 143 | 61 |  | 98 | 42 |  | | 50 | | 21 |
| 11-10 | 189 | 80 |  | 146 | 62 |  | 97 | 41 |  | | 48 | | 20 |
| 11-12 | 236 | 100 |  | 236 | 100 |  | 230 | 97 |  | | 214 | | 91 |
| 11-13 | 236 | 100 |  | 234 | 99 |  | 233 | 99 |  | | 221 | | 94 |
| 12-1 | 207 | 88 |  | 177 | 75 |  | 130 | 55 |  | | 69 | | 29 |
| 12-2 | 200 | 85 |  | 162 | 69 |  | 104 | 44 |  | | 59 | | 25 |
| 12-3 | 229 | 97 |  | 218 | 92 |  | 199 | 84 |  | | 152 | | 64 |
| 12-4 | 230 | 97 |  | 217 | 92 |  | 193 | 82 |  | | 146 | | 62 |
| 12-5 | 234 | 99 |  | 229 | 97 |  | 218 | 92 |  | | 166 | | 70 |
| 12-6 | 202 | 86 |  | 164 | 69 |  | 112 | 47 |  | | 61 | | 26 |
| 12-7 | 229 | 97 |  | 213 | 90 |  | 176 | 75 |  | | 127 | | 54 |
| 12-8 | 204 | 86 |  | 179 | 76 |  | 119 | 50 |  | | 71 | | 30 |
| 12-9 | 236 | 100 |  | 235 | 100 |  | 233 | 99 |  | | 216 | | 92 |
| 12-10 | 236 | 100 |  | 235 | 100 |  | 232 | 98 |  | | 218 | | 92 |
| 12-11 | 235 | 100 |  | 233 | 99 |  | 225 | 95 |  | | 205 | | 87 |
| 12-13 | 27 | 11 |  | 6 | 3 |  | 5 | 2 |  | | 2 | | 1 |
| 13-1 | 209 | 89 |  | 180 | 76 |  | 134 | 57 |  | | 70 | | 30 |
| 13-2 | 203 | 86 |  | 163 | 69 |  | 103 | 44 |  | | 68 | | 29 |
| 13-3 | 230 | 97 |  | 217 | 92 |  | 201 | 85 |  | | 146 | | 62 |
| 13-4 | 230 | 97 |  | 219 | 93 |  | 197 | 83 |  | | 147 | | 62 |
| 13-5 | 234 | 99 |  | 230 | 97 |  | 219 | 93 |  | | 183 | | 78 |
| 13-6 | 205 | 87 |  | 165 | 70 |  | 114 | 48 |  | | 64 | | 27 |
| 13-7 | 227 | 96 |  | 213 | 90 |  | 177 | 75 |  | | 132 | | 56 |
| 13-8 | 207 | 88 |  | 177 | 75 |  | 125 | 53 |  | | 69 | | 29 |
| 13-9 | 236 | 100 |  | 235 | 100 |  | 231 | 98 |  | | 210 | | 89 |
| 13-10 | 236 | 100 |  | 235 | 100 |  | 234 | 99 |  | | 228 | | 97 |
| 13-11 | 235 | 100 |  | 233 | 99 |  | 225 | 95 |  | | 211 | | 89 |
| 13-12 | 26 | 11 |  | 5 | 2 |  | 5 | 2 |  | | 2 | | 1 |
