# Supplementary material for: The power of phylogenetic approaches to detect horizontally transferred genes
Source: BMC Evol Biol. 2007 Mar 21;7:45. doi: 10.1186/1471-2148-7-45 (PMC1847511; doi:10.1186/1471-2148-7-45)
Supplement: Additional file 2 — Power of HGT detection using the symmetric difference of Robinson and Foulds distance. Complementary tables for Figure 6. [file 1471-2148-7-45-S2.doc]

**Power of HGT detection using the symmetric difference**

**of Robinson and Foulds distance.**

**Complementary tables for Figure 6.**

**Table1. Symmetric difference of Robinson and Foulds. Gene exchange between species.**

**Complementary Tables for Figure 6A.**

*Nu – is the number of families that showed significant conflict at a given significance level, % - relative number of families. Total number of families is 236.*

**A. Ordered by the position in the tree**

| **Symmetric Difference. Gene Exchange Between Species.**  *Significance level 2σ; values in %.* | | | | | | | | | | | | | | |
| --- | --- | --- | --- | --- | --- | --- | --- | --- | --- | --- | --- | --- | --- | --- |
|  |  | **5** | **7** | **8** | **1** | **12** | **13** | **6** | **2** | **4** | **3** | **11** | **9** | **10** |
| **/--------------** | **5** | 0 |  |  |  |  |  |  |  |  |  |  |  |  |
| **| /--------** | **7** | 3 | 0 |  |  |  |  |  |  |  |  |  |  |  |
| **| | ____/--** | **8** | 33 | 10 | 0 |  |  |  |  |  |  |  |  |  |  |
| **|_____| | \--** | **1** | 32 | 10 | 3 | 0 |  |  |  |  |  |  |  |  |  |
| **| | |_| __/--** | **12** | 100 | 26 | 45 | 45 | 0 |  |  |  |  |  |  |  |  |
| **| | |_/ \--** | **13** | 100 | 26 | 45 | 45 | 3 | 0 |  |  |  |  |  |  |  |
| **| | \__/--** | **6** | 100 | 23 | 44 | 44 | 19 | 19 | 0 |  |  |  |  |  |  |
| **| | \--** | **2** | 100 | 23 | 44 | 44 | 19 | 19 | 3 | 0 |  |  |  |  |  |
| **| |________/--** | **4** | 17 | 6 | 31 | 31 | 65 | 65 | 64 | 64 | 0 |  |  |  |  |
| **| \--** | **3** | 17 | 6 | 31 | 31 | 65 | 65 | 64 | 64 | 3 | 0 |  |  |  |
| **|__________/----** | **11** | 5 | 13 | 100 | 100 | 100 | 100 | 100 | 100 | 70 | 70 | 0 |  |  |
| **\_/--** | **9** | 19 | 51 | 100 | 100 | 100 | 100 | 100 | 100 | 100 | 100 | 5 | 0 |  |
| **\--** | **10** | 19 | 51 | 100 | 100 | 100 | 100 | 100 | 100 | 100 | 100 | 4 | 3 | 0 |

**B. Ordered by species’ numbers.**

|  | **Significance level** | |
| --- | --- | --- |
|  | **2σ** | |
| **flip** | **Nu** | **%** |
| 1-2 | 103 | 44 |
| 1-3 | 73 | 31 |
| 1-4 | 73 | 31 |
| 1-5 | 76 | 32 |
| 1-6 | 103 | 44 |
| 1-7 | 23 | 10 |
| 1-8 | 6 | 3 |
| 1-9 | 235 | 100 |
| 1-10 | 235 | 100 |
| 1-11 | 235 | 100 |
| 1-12 | 107 | 45 |
| 1-13 | 107 | 45 |
| 2-3 | 151 | 64 |
| 2-4 | 151 | 64 |
| 2-5 | 235 | 100 |
| 2-6 | 6 | 3 |
| 2-7 | 55 | 23 |
| 2-8 | 103 | 44 |
| 2-9 | 235 | 100 |
| 2-10 | 235 | 100 |
| 2-11 | 235 | 100 |
| 2-12 | 45 | 19 |
| 2-13 | 44 | 19 |
| 3-4 | 6 | 3 |
| 3-5 | 40 | 17 |
| 3-6 | 151 | 64 |
| 3-7 | 13 | 6 |
| 3-8 | 73 | 31 |
| 3-9 | 235 | 100 |
| 3-10 | 235 | 100 |
| 3-11 | 165 | 70 |
| 3-12 | 153 | 65 |
| 3-13 | 153 | 65 |
| 4-5 | 40 | 17 |
| 4-6 | 151 | 64 |
| 4-7 | 13 | 6 |
| 4-8 | 73 | 31 |
| 4-9 | 235 | 100 |
| 4-10 | 235 | 100 |
| 4-11 | 165 | 70 |
| 4-12 | 153 | 65 |
| 4-13 | 153 | 65 |
| 5-6 | 235 | 100 |
| 5-7 | 8 | 3 |
| 5-8 | 77 | 33 |
| 5-9 | 45 | 19 |
| 5-10 | 45 | 19 |
| 5-11 | 12 | 5 |
| 5-12 | 235 | 100 |
| 5-13 | 235 | 100 |
| 6-7 | 55 | 23 |
| 6-8 | 103 | 44 |
| 6-9 | 235 | 100 |
| 6-10 | 235 | 100 |
| 6-11 | 235 | 100 |
| 6-12 | 44 | 19 |
| 6-13 | 45 | 19 |
| 7-8 | 24 | 10 |
| 7-9 | 121 | 51 |
| 7-10 | 121 | 51 |
| 7-11 | 30 | 13 |
| 7-12 | 61 | 26 |
| 7-13 | 61 | 26 |
| 8-9 | 235 | 100 |
| 8-10 | 235 | 100 |
| 8-11 | 235 | 100 |
| 8-12 | 107 | 45 |
| 8-13 | 107 | 45 |
| 9-10 | 6 | 3 |
| 9-11 | 11 | 5 |
| 9-12 | 235 | 100 |
| 9-13 | 235 | 100 |
| 10-11 | 10 | 4 |
| 10-12 | 235 | 100 |
| 10-13 | 235 | 100 |
| 11-12 | 235 | 100 |
| 11-13 | 235 | 100 |
| 12-13 | 6 | 3 |

**Table 2. Symmetric difference of Robinson and Foulds. Gene donation with replacement.**

**Complementary tables for figure 6B.**

*Nu – is the number of families that showed significant conflict at a given significance level, % - relative number of families. Total number of families is 236.*

**A. Ordered by the position on the tree**

| **Symmetric Difference. Gene Donation With Replacement.**  *Significance level 2σ; values in %.* | | | | | | | | | | | | | | |
| --- | --- | --- | --- | --- | --- | --- | --- | --- | --- | --- | --- | --- | --- | --- |
|  |  | **5** | **7** | **8** | **1** | **12** | **13** | **6** | **2** | **4** | **3** | **11** | **9** | **10** |
| **/--------------** | **5** | 0 | 3 | 33 | 33 | 100 | 100 | 100 | 100 | 17 | 17 | 5 | 19 | 19 |
| **| /--------** | **7** | 3 | 0 | 11 | 10 | 26 | 26 | 23 | 23 | 6 | 6 | 11 | 51 | 51 |
| **| | ____/--** | **8** | 33 | 8 | 0 | 1 | 45 | 45 | 44 | 44 | 31 | 31 | 100 | 100 | 100 |
| **|_____| | \--** | **1** | 33 | 8 | 1 | 0 | 45 | 45 | 44 | 44 | 31 | 31 | 100 | 100 | 100 |
| **| | |_| __/--** | **12** | 100 | 22 | 45 | 45 | 0 | 2 | 19 | 19 | 64 | 65 | 100 | 100 | 100 |
| **| | |_/ \--** | **13** | 100 | 22 | 45 | 45 | 2 | 0 | 19 | 19 | 64 | 65 | 100 | 100 | 100 |
| **| | \__/--** | **6** | 100 | 20 | 43 | 44 | 19 | 19 | 0 | 1 | 64 | 64 | 100 | 100 | 100 |
| **| | \--** | **2** | 100 | 20 | 43 | 44 | 19 | 19 | 1 | 0 | 64 | 64 | 100 | 100 | 100 |
| **| |________/--** | **4** | 17 | 5 | 31 | 31 | 65 | 65 | 64 | 64 | 0 | 2 | 69 | 100 | 100 |
| **| \--** | **3** | 17 | 5 | 31 | 31 | 65 | 65 | 64 | 64 | 3 | 0 | 69 | 100 | 100 |
| **|__________/----** | **11** | 3 | 6 | 100 | 100 | 100 | 100 | 100 | 100 | 70 | 70 | 0 | 5 | 5 |
| **\_/--** | **9** | 11 | 21 | 100 | 100 | 100 | 100 | 100 | 100 | 100 | 100 | 5 | 0 | 2 |
| **\--** | **10** | 11 | 21 | 100 | 100 | 100 | 100 | 100 | 100 | 100 | 100 | 5 | 2 | 0 |

**B. Ordered by species’ numbers.**

|  | **Significance level** | |
| --- | --- | --- |
| **donate** | **2σ** | |
|  | **Nu** | **%** |
| 1-2 | 103 | 44 |
| 1-3 | 72 | 31 |
| 1-4 | 73 | 31 |
| 1-5 | 77 | 33 |
| 1-6 | 103 | 44 |
| 1-7 | 19 | 8 |
| 1-8 | 2 | 1 |
| 1-9 | 235 | 100 |
| 1-10 | 235 | 100 |
| 1-11 | 235 | 100 |
| 1-12 | 107 | 45 |
| 1-13 | 107 | 45 |
| 2-1 | 103 | 44 |
| 2-3 | 151 | 64 |
| 2-4 | 150 | 64 |
| 2-5 | 235 | 100 |
| 2-6 | 2 | 1 |
| 2-7 | 47 | 20 |
| 2-8 | 102 | 43 |
| 2-9 | 235 | 100 |
| 2-10 | 235 | 100 |
| 2-11 | 235 | 100 |
| 2-12 | 45 | 19 |
| 2-13 | 45 | 19 |
| 3-1 | 73 | 31 |
| 3-2 | 151 | 64 |
| 3-4 | 6 | 3 |
| 3-5 | 40 | 17 |
| 3-6 | 151 | 64 |
| 3-7 | 12 | 5 |
| 3-8 | 73 | 31 |
| 3-9 | 235 | 100 |
| 3-10 | 235 | 100 |
| 3-11 | 164 | 69 |
| 3-12 | 153 | 65 |
| 3-13 | 153 | 65 |
| 4-1 | 73 | 31 |
| 4-2 | 151 | 64 |
| 4-3 | 5 | 2 |
| 4-5 | 40 | 17 |
| 4-6 | 151 | 64 |
| 4-7 | 12 | 5 |
| 4-8 | 73 | 31 |
| 4-9 | 235 | 100 |
| 4-10 | 235 | 100 |
| 4-11 | 164 | 69 |
| 4-12 | 153 | 65 |
| 4-13 | 153 | 65 |
| 5-1 | 77 | 33 |
| 5-2 | 235 | 100 |
| 5-3 | 40 | 17 |
| 5-4 | 40 | 17 |
| 5-6 | 235 | 100 |
| 5-7 | 6 | 3 |
| 5-8 | 77 | 33 |
| 5-9 | 45 | 19 |
| 5-10 | 45 | 19 |
| 5-11 | 11 | 5 |
| 5-12 | 235 | 100 |
| 5-13 | 235 | 100 |
| 6-1 | 103 | 44 |
| 6-2 | 2 | 1 |
| 6-3 | 151 | 64 |
| 6-4 | 150 | 64 |
| 6-5 | 235 | 100 |
| 6-7 | 47 | 20 |
| 6-8 | 102 | 43 |
| 6-9 | 235 | 100 |
| 6-10 | 235 | 100 |
| 6-11 | 235 | 100 |
| 6-12 | 45 | 19 |
| 6-13 | 45 | 19 |
| 7-1 | 24 | 10 |
| 7-2 | 55 | 23 |
| 7-3 | 13 | 6 |
| 7-4 | 13 | 6 |
| 7-5 | 8 | 3 |
| 7-6 | 55 | 23 |
| 7-8 | 25 | 11 |
| 7-9 | 121 | 51 |
| 7-10 | 121 | 51 |
| 7-11 | 26 | 11 |
| 7-12 | 61 | 26 |
| 7-13 | 61 | 26 |
| 8-1 | 2 | 1 |
| 8-2 | 103 | 44 |
| 8-3 | 72 | 31 |
| 8-4 | 73 | 31 |
| 8-5 | 77 | 33 |
| 8-6 | 103 | 44 |
| 8-7 | 19 | 8 |
| 8-9 | 235 | 100 |
| 8-10 | 235 | 100 |
| 8-11 | 235 | 100 |
| 8-12 | 107 | 45 |
| 8-13 | 107 | 45 |
| 9-1 | 235 | 100 |
| 9-2 | 235 | 100 |
| 9-3 | 235 | 100 |
| 9-4 | 235 | 100 |
| 9-5 | 26 | 11 |
| 9-6 | 235 | 100 |
| 9-7 | 49 | 21 |
| 9-8 | 235 | 100 |
| 9-10 | 5 | 2 |
| 9-11 | 11 | 5 |
| 9-12 | 235 | 100 |
| 9-13 | 235 | 100 |
| 10-1 | 235 | 100 |
| 10-2 | 235 | 100 |
| 10-3 | 235 | 100 |
| 10-4 | 235 | 100 |
| 10-5 | 26 | 11 |
| 10-6 | 235 | 100 |
| 10-7 | 49 | 21 |
| 10-8 | 235 | 100 |
| 10-9 | 5 | 2 |
| 10-11 | 11 | 5 |
| 10-12 | 235 | 100 |
| 10-13 | 235 | 100 |
| 11-1 | 235 | 100 |
| 11-2 | 235 | 100 |
| 11-3 | 165 | 70 |
| 11-4 | 165 | 70 |
| 11-5 | 8 | 3 |
| 11-6 | 235 | 100 |
| 11-7 | 13 | 6 |
| 11-8 | 235 | 100 |
| 11-9 | 11 | 5 |
| 11-10 | 11 | 5 |
| 11-12 | 235 | 100 |
| 11-13 | 235 | 100 |
| 12-1 | 107 | 45 |
| 12-2 | 45 | 19 |
| 12-3 | 153 | 65 |
| 12-4 | 152 | 64 |
| 12-5 | 235 | 100 |
| 12-6 | 45 | 19 |
| 12-7 | 53 | 22 |
| 12-8 | 106 | 45 |
| 12-9 | 235 | 100 |
| 12-10 | 235 | 100 |
| 12-11 | 235 | 100 |
| 12-13 | 5 | 2 |
| 13-1 | 107 | 45 |
| 13-2 | 45 | 19 |
| 13-3 | 153 | 65 |
| 13-4 | 152 | 64 |
| 13-5 | 235 | 100 |
| 13-6 | 45 | 19 |
| 13-7 | 53 | 22 |
| 13-8 | 106 | 45 |
| 13-9 | 235 | 100 |
| 13-10 | 235 | 100 |
| 13-11 | 235 | 100 |
| 13-12 | 5 | 2 |
