# Supplementary material for: The power of phylogenetic approaches to detect horizontally transferred genes
Source: BMC Evol Biol. 2007 Mar 21;7:45. doi: 10.1186/1471-2148-7-45 (PMC1847511; doi:10.1186/1471-2148-7-45)
Supplement: Additional file 3 — Power of HGT detection using bipartition spectra on genome data with in silico transfer. Complementary tables for Figure 8. [file 1471-2148-7-45-S3.doc]

**Power of HGT detection using bipartition spectra on genome data**

**with *in silico* transfer.**

**Complementary tables for Figure 8.**

**Table 1. Bipartitions. Gene exchange between species**

*Nu – is the number of families that showed significant conflict at a given significance level, % - relative number of families. Total number of families is 236.*

*80% is included into the table but is not depicted on Figure 8.*

1. **Ordered by the position in the tree**

| **Bipartitions. Gene Exchange Between Species.**  *Blue - Bootstrap support 70%; Green – Bootstrap support 90%; values in %.* | | | | | | | | | | | | | | |
| --- | --- | --- | --- | --- | --- | --- | --- | --- | --- | --- | --- | --- | --- | --- |
|  |  | **5** | **7** | **8** | **1** | **12** | **13** | **6** | **2** | **4** | **3** | **11** | **9** | **10** |
| **/--------------** | **5** | 0 | 81 | 94 | 94 | 99 | 99 | 96 | 96 | 98 | 98 | 96 | 99 | 99 |
| **| /--------** | **7** | 67 | 0 | 91 | 90 | 99 | 99 | 94 | 94 | 97 | 97 | 94 | 99 | 99 |
| **| | ____/--** | **8** | 89 | 82 | 0 | 7 | 98 | 98 | 96 | 96 | 97 | 97 | 97 | 98 | 98 |
| **|_____| | \--** | **1** | 89 | 82 | 2 | 0 | 98 | 98 | 96 | 96 | 97 | 97 | 97 | 98 | 98 |
| **| | |_| __/--** | **12** | 97 | 97 | 96 | 96 | 0 | 7 | 97 | 97 | 99 | 99 | 97 | 99 | 99 |
| **| | |_/ \--** | **13** | 97 | 97 | 96 | 96 | 2 | 0 | 97 | 97 | 99 | 99 | 97 | 99 | 99 |
| **| | \__/--** | **6** | 91 | 86 | 93 | 93 | 94 | 94 | 0 | 7 | 98 | 98 | 98 | 98 | 98 |
| **| | \--** | **2** | 91 | 86 | 93 | 93 | 94 | 94 | 2 | 0 | 98 | 98 | 98 | 98 | 98 |
| **| |________/--** | **4** | 94 | 88 | 94 | 94 | 98 | 98 | 92 | 92 | 0 | 7 | 99 | 99 | 99 |
| **| \--** | **3** | 94 | 88 | 94 | 94 | 98 | 98 | 92 | 92 | 2 | 0 | 99 | 99 | 99 |
| **|__________/----** | **11** | 98 | 97 | 97 | 97 | 99 | 99 | 96 | 96 | 99 | 99 | 0 | 92 | 92 |
| **\_/--** | **9** | 98 | 98 | 97 | 97 | 99 | 99 | 96 | 96 | 99 | 99 | 79 | 0 | 7 |
| **\--** | **10** | 98 | 98 | 97 | 97 | 99 | 99 | 96 | 96 | 99 | 99 | 79 | 2 | 0 |

**B. Ordered by species’ numbers.**

|  | **Bootstrap support** | |  | **Bootstrap support** | |  | **Bootstrap support** | |
| --- | --- | --- | --- | --- | --- | --- | --- | --- |
| flip | 70% | |  | 80% | |  | 90% | |
|  | **Nu** | **%** |  | **Nu** | **%** |  | **Nu** | **%** |
| 1-2 | 226 | 96 |  | 222 | 94 |  | 220 | 93 |
| 1-3 | 230 | 97 |  | 227 | 96 |  | 222 | 94 |
| 1-4 | 230 | 97 |  | 227 | 96 |  | 222 | 94 |
| 1-5 | 221 | 94 |  | 213 | 90 |  | 209 | 89 |
| 1-6 | 226 | 96 |  | 222 | 94 |  | 220 | 93 |
| 1-7 | 213 | 90 |  | 202 | 86 |  | 193 | 82 |
| 1-8 | 17 | 7 |  | 10 | 4 |  | 5 | 2 |
| 1-9 | 231 | 98 |  | 230 | 97 |  | 230 | 97 |
| 1-10 | 231 | 98 |  | 230 | 97 |  | 230 | 97 |
| 1-11 | 230 | 97 |  | 229 | 97 |  | 229 | 97 |
| 1-12 | 231 | 98 |  | 229 | 97 |  | 227 | 96 |
| 1-13 | 231 | 98 |  | 229 | 97 |  | 227 | 96 |
| 2-3 | 231 | 98 |  | 220 | 93 |  | 218 | 92 |
| 2-4 | 231 | 98 |  | 220 | 93 |  | 218 | 92 |
| 2-5 | 227 | 96 |  | 218 | 92 |  | 214 | 91 |
| 2-6 | 17 | 7 |  | 10 | 4 |  | 5 | 2 |
| 2-7 | 223 | 94 |  | 209 | 89 |  | 202 | 86 |
| 2-8 | 226 | 96 |  | 222 | 94 |  | 220 | 93 |
| 2-9 | 231 | 98 |  | 227 | 96 |  | 227 | 96 |
| 2-10 | 231 | 98 |  | 227 | 96 |  | 227 | 96 |
| 2-11 | 231 | 98 |  | 227 | 96 |  | 227 | 96 |
| 2-12 | 230 | 97 |  | 224 | 95 |  | 223 | 94 |
| 2-13 | 230 | 97 |  | 224 | 95 |  | 223 | 94 |
| 3-4 | 17 | 7 |  | 10 | 4 |  | 5 | 2 |
| 3-5 | 232 | 98 |  | 228 | 97 |  | 221 | 94 |
| 3-6 | 231 | 98 |  | 220 | 93 |  | 218 | 92 |
| 3-7 | 229 | 97 |  | 216 | 92 |  | 207 | 88 |
| 3-8 | 230 | 97 |  | 227 | 96 |  | 222 | 94 |
| 3-9 | 234 | 99 |  | 234 | 99 |  | 234 | 99 |
| 3-10 | 234 | 99 |  | 234 | 99 |  | 234 | 99 |
| 3-11 | 234 | 99 |  | 234 | 99 |  | 234 | 99 |
| 3-12 | 234 | 99 |  | 233 | 99 |  | 232 | 98 |
| 3-13 | 234 | 99 |  | 233 | 99 |  | 232 | 98 |
| 4-5 | 232 | 98 |  | 228 | 97 |  | 221 | 94 |
| 4-6 | 231 | 98 |  | 220 | 93 |  | 218 | 92 |
| 4-7 | 230 | 97 |  | 216 | 92 |  | 207 | 88 |
| 4-8 | 230 | 97 |  | 227 | 96 |  | 222 | 94 |
| 4-9 | 234 | 99 |  | 234 | 99 |  | 234 | 99 |
| 4-10 | 234 | 99 |  | 234 | 99 |  | 234 | 99 |
| 4-11 | 234 | 99 |  | 234 | 99 |  | 234 | 99 |
| 4-12 | 234 | 99 |  | 233 | 99 |  | 232 | 98 |
| 4-13 | 234 | 99 |  | 233 | 99 |  | 232 | 98 |
| 5-6 | 227 | 96 |  | 218 | 92 |  | 214 | 91 |
| 5-7 | 190 | 81 |  | 171 | 72 |  | 157 | 67 |
| 5-8 | 221 | 94 |  | 213 | 90 |  | 209 | 89 |
| 5-9 | 233 | 99 |  | 231 | 98 |  | 232 | 98 |
| 5-10 | 233 | 99 |  | 231 | 98 |  | 232 | 98 |
| 5-11 | 227 | 96 |  | 229 | 97 |  | 231 | 98 |
| 5-12 | 234 | 99 |  | 232 | 98 |  | 230 | 97 |
| 5-13 | 234 | 99 |  | 232 | 98 |  | 230 | 97 |
| 6-7 | 223 | 94 |  | 209 | 89 |  | 202 | 86 |
| 6-8 | 226 | 96 |  | 222 | 94 |  | 220 | 93 |
| 6-9 | 231 | 98 |  | 227 | 96 |  | 227 | 96 |
| 6-10 | 231 | 98 |  | 227 | 96 |  | 227 | 96 |
| 6-11 | 231 | 98 |  | 227 | 96 |  | 227 | 96 |
| 6-12 | 230 | 97 |  | 224 | 95 |  | 223 | 94 |
| 6-13 | 230 | 97 |  | 224 | 95 |  | 223 | 94 |
| 7-8 | 214 | 91 |  | 203 | 86 |  | 193 | 82 |
| 7-9 | 233 | 99 |  | 231 | 98 |  | 232 | 98 |
| 7-10 | 233 | 99 |  | 231 | 98 |  | 232 | 98 |
| 7-11 | 223 | 94 |  | 226 | 96 |  | 228 | 97 |
| 7-12 | 234 | 99 |  | 232 | 98 |  | 230 | 97 |
| 7-13 | 234 | 99 |  | 232 | 98 |  | 230 | 97 |
| 8-9 | 231 | 98 |  | 230 | 97 |  | 230 | 97 |
| 8-10 | 231 | 98 |  | 230 | 97 |  | 230 | 97 |
| 8-11 | 230 | 97 |  | 229 | 97 |  | 229 | 97 |
| 8-12 | 231 | 98 |  | 229 | 97 |  | 227 | 96 |
| 8-13 | 231 | 98 |  | 229 | 97 |  | 227 | 96 |
| 9-10 | 17 | 7 |  | 10 | 4 |  | 5 | 2 |
| 9-11 | 218 | 92 |  | 196 | 83 |  | 187 | 79 |
| 9-12 | 234 | 99 |  | 233 | 99 |  | 233 | 99 |
| 9-13 | 234 | 99 |  | 233 | 99 |  | 233 | 99 |
| 10-11 | 217 | 92 |  | 197 | 83 |  | 187 | 79 |
| 10-12 | 234 | 99 |  | 233 | 99 |  | 233 | 99 |
| 10-13 | 234 | 99 |  | 233 | 99 |  | 233 | 99 |
| 11-12 | 230 | 97 |  | 233 | 99 |  | 234 | 99 |
| 11-13 | 230 | 97 |  | 233 | 99 |  | 234 | 99 |
| 12-13 | 17 | 7 |  | 10 | 4 |  | 5 | 2 |
